# TREM2 and sTREM2 in Alzheimer’s disease: from mechanisms to therapies

**DOI:** 10.1186/s13024-025-00834-z

**Published:** 2025-04-17

**Authors:** Lianshuai Zhang, Xianyuan Xiang, Yahui Li, Guojun Bu, Xiao-Fen Chen

**Affiliations:** 1 State Key Laboratory of Cellular Stress Biology, Fujian Provincial Key Laboratory of Neurodegenerative Disease and Aging Research, Institute of Neuroscience, School of Medicine, Xiamen University, Xiamen, Fujian 361102 China; 2 Shenzhen Research Institute of Xiamen University, Shenzhen, 518057 China; 3 The Brain Cognition and Brain Disease Institute, Shenzhen Institutes of Advanced Technology, Chinese Academy of Sciences, Shenzhen, 518055 China; 4 Faculty of Life and Health Sciences, Shenzhen University of Advanced Technology, Shenzhen, 518055 China; 5 Division of Life Science and State Key Laboratory of Molecular Neuroscience, The Hong Kong University of Science and Technology, Hong Kong, China

**Keywords:** TREM2, sTREM2, Microglia, Metabolism, Amyloid, Tau, Neurodegeneration, Alzheimer’s disease

## Abstract

Triggering receptor expressed on myeloid cells 2 (TREM2) is an innate immune receptor predominantly expressed by microglia in the brain. Recent studies have established TREM2 as a central immune signaling hub in neurodegeneration, where it triggers immune responses upon sensing pathological development and tissue damages. TREM2 binds diverse ligands and activates downstream pathways that regulate microglial phagocytosis, inflammatory responses, and metabolic reprogramming. Interestingly, TREM2 exists both in its membrane-bound form and as a soluble variant (sTREM2), that latter is generated through proteolytic shedding or alternative splicing and can be detected in cerebrospinal fluid and plasma. Emerging clinical and preclinical evidence underscores the potential of TREM2 and sTREM2 as diagnostic biomarkers and therapeutic targets in Alzheimer’s disease (AD). This review provides a comprehensive overview of the molecular functions, regulatory mechanisms, and pathological implications of TREM2 and sTREM2 in AD. Furthermore, we explore their potential roles in diagnostics and therapeutics while suggesting key research directions for advancing TREM2/sTREM2-based strategies in combating AD.

## Background

Alzheimer’s disease (AD) is a devastating neurodegenerative disorder marked by progressive cognitive decline and key neuropathological features, such as amyloid-β (Aβ) plaques, neurofibrillary tangles, and chronic neuroinflammation [1–3]. While significant strides have been made in understanding these pathological markers, the role of microglia—the resident immune cells of the central nervous system (CNS)—has gained increasing attention over the past decade. Microglia play a crucial role in maintaining brain homeostasis; however, they become dysregulated in AD, contributing to disease progression through maladaptive neuroinflammatory responses and impaired clearance of neurotoxic materials [4, 5]. A key player in microglial functions is the triggering receptor expressed on myeloid cells 2 (TREM2), a transmembrane receptor that has been strongly associated with the risk for AD [6, 7]. TREM2 has emerged as a critical modulator in shaping microglial responses to AD, making it a compelling therapeutic target for mitigating AD-associated pathology [8–10].

TREM2 signaling is mediated through its interaction with adaptor proteins DNAX-activating protein 12 (DAP12) and DAP10, which contain essential motifs for signal transduction [11]. Binding of ligands—including lipids, lipoproteins, apolipoproteins, or Aβ—to TREM2 triggers intracellular signaling cascades that regulate microglial responses to AD pathology [10, 12]. Notably, genetic variants of TREM2, especially the R47H mutation, are strongly associated with increased AD risk [6, 7]. These mutations impair TREM2 functions, leading to reduced microglial activation, dysregulated inflammatory responses, impaired metabolic functions, and inadequate clearance of Aβ. Collectively, these factors exacerbate AD pathology, emphasizing TREM2’s central role in both AD susceptibility and progression, and reinforcing its potential as a therapeutic target to enhance microglial protective function and combat disease mechanisms [13].

Beyond its role as a cell-surface receptor, TREM2 undergoes proteolytic cleavage or alternative splicing, resulting in the release of its soluble ectodomain (sTREM2) into the extracellular space, where it can be detected in cerebrospinal fluid (CSF) and plasma [14–17]. As both a biomarker and a potential modulator in AD, sTREM2 has been associated with microglial activation and disease progression, as evidenced by findings from human studies and AD mouse models. Elevated CSF sTREM2 levels correlate with early AD stages and neuroinflammatory responses, suggesting its potential as a diagnostic biomarker [18, 19]. Furthermore, preclinical and clinical studies indicate that sTREM2 may exert neuroprotective effects by mitigating Aβ and tau pathologies, potentially slowing cognitive decline and disease progression [20–23].

Despite these advances, many questions remain unanswered regarding the precise mechanisms by which TREM2 and sTREM2 influence microglial functions and AD pathogenesis. Understanding the interplay between genetic variations, TREM2 signaling, and sTREM2-mediated effects could provide valuable insights into AD pathogenesis and guide the development of targeted therapies. This review aims to synthesize current knowledge on TREM2 and sTREM2 in AD, focusing on their molecular functions, pathological implications, and therapeutic potential. By consolidating recent findings, we seek to highlight critical gaps in our understanding and propose future directions for advancing TREM2/sTREM2-based strategies in AD diagnostics and therapeutics.

## TREM2 as a cell-surface receptor

### TREM2 genetic variants and signaling

Genetic variations in TREM2 have been implicated in several neurodegenerative disorders, including AD, frontotemporal dementia (FTD), and Nasu-Hakola disease (NHD), with over 70 variants identified to date [9]. Homozygous TREM2 mutations, including early stop codons (E14X, Q33X, W44X, W78X) [24–27] and missense mutations in the immunoglobulin-like ectodomain (Y38C, W50C, T66M, V126G) [24, 28, 29], are primarily linked to NHD, a rare disorder characterized by early-onset dementia, demyelination, and bone cysts. Notably, some patients carrying specific homozygous TREM2 mutations, such as Q33X, Y38C, and T66M, exhibit an FTD-like syndrome with behavioral changes, cognitive decline, and motor impairments [24]. Unlike classical NHD, these FTD patients do not develop bone cysts or other obvious skeletal abnormalities. This phenotypic variability suggests that the clinical manifestations of these mutations may also be influenced by genetic background or environmental factors.

The most well-established TREM2 variant associated with AD is the R47H substitution (rs75932628) [6, 7, 30]. Early studies in European and North American cohorts identified a strong association between heterozygous R47H carriers and AD, reporting odds ratios (ORs) ranging from 2.83 to 4.59—comparable to the risk conferred by the APOE ε4 allele [6, 7]. These findings have been consistently validated by subsequent meta-analyses and replication studies [31–36]. Notably, individuals homozygous for the R47H variant face an even greater risk of developing AD compared to heterozygous carriers [30]. Interestingly, cognitive decline has been observed in R47H carriers between the ages of 80 and 100, even in the absence of an AD diagnosis [7]. However, the association between R47H and AD appears weaker in non-European populations, including African-American [37], Chinese [38–41], and Japanese [42] cohorts. Furthermore, while R47H has been linked to an increased risk of FTD in North American populations [43], no such association has been observed in European cohorts [36], highlighting the geographic and genetic heterogeneity in its effects.

Other AD-associated TREM2 variants also exhibit population-specific associations. For instance, the R62H variant (rs143332484) serves as a significant AD risk modifier in Caucasian populations [34, 35], but it is not associated with AD risk in African-Americans, likely due to its low frequency in this population [37]. The H157Y variant (rs2234255), identified in Han Chinese populations, is linked to late-onset AD [44], with subsequent studies supporting the association in the Alzheimer’s Disease Sequencing Project (ORs: 4.7) [45], while a large Caucasian case-control study found no significant correlation [35]. These findings highlight the intricate role of TREM2 genetic variations in neurodegeneration, with their effects influenced by mutation type, genetic ancestry, and disease stage or pathological environment. The three-dimensional structure of TREM2, elucidated by X-ray crystallography, offers further insights into the functional consequences of these genetic variants. NHD-associated mutations are predominantly localized to buried regions of the protein, potentially destabilizing its structure [46]. In contrast, AD-associated variants reside on the protein’s surface, impairing ligand binding and receptor activation. For example, the R47H substitution reduces TREM2’s affinity for key ligands, including phospholipids and Aβ, leading to diminished downstream signaling [47–49].

**Fig. 1:**
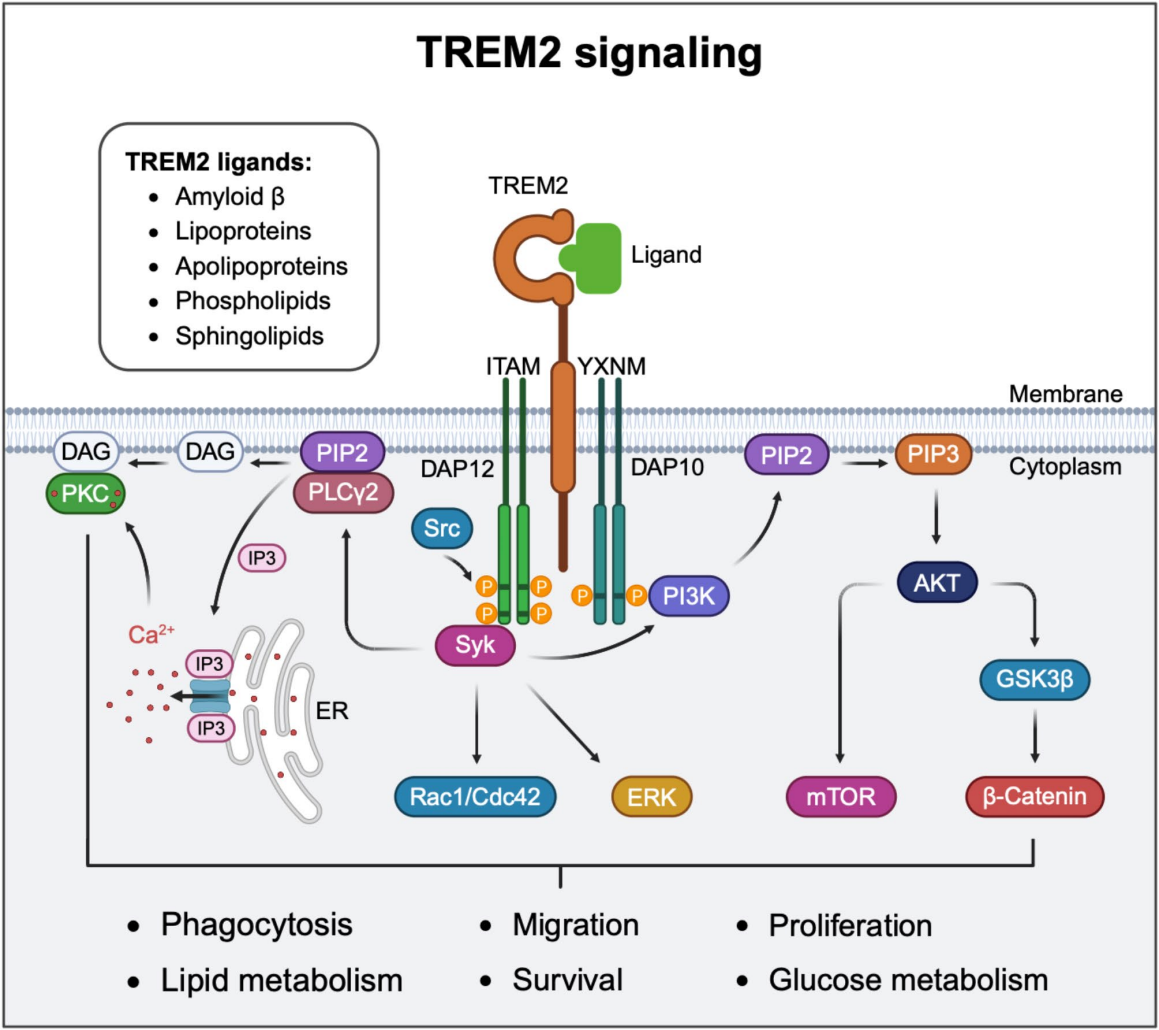
Schematic illustration of TREM2 signaling. The membrane-bound TREM2 receptor interacts with various ligands, including lipids, lipoproteins, apolipoproteins and amyloid-β. Ligand binding to TREM2 triggers the phosphorylation of tyrosine residues within the ITAM motif of the DAP12 cytoplasmic domain by SRC family kinases. The phosphorylated ITAM recruit the protein tyrosine kinase SYK to activate downstream signaling pathways. Additionally, TREM2 can associate with DAP10 homodimer, which contains YXNM motifs. These motifs directly recruit PI3K, activating further signaling pathways. SYK activation drives key pathways, including PLCγ2, Rac1/Cdc42, ERK, and PI3K. PLCγ2 activation is critical for microglial phagocytosis and lipid metabolism. Meanwhile, Rac1/Cdc42-GTPase signaling plays a key role in cytoskeletal remodeling and cell migration. Additionally, the ERK pathway is essential for microglial survival, proliferation, and inflammatory responses. In parallel, the PI3K/Akt pathway, activated by both DAP10 and SYK, governs essential cellular processes such as survival, proliferation, and glucose metabolism. PIP3, phosphatidylinositol 3,4,5-trisphosphate; PIP2, phosphatidylinositol (4,5)-bisphosphate; PLCγ2, phospholipase Cγ2; DAG, diacylglycerol; IP3, inositol 1,4,5-trisphosphate; PKC, protein kinase C; PI3K, phosphatidylinositol 3-kinase; ERK, extracellular signal-regulated kinase; GSK3β, glycogen synthase kinase 3β; mTOR, mechanistic target of rapamycin

TREM2 signaling is mediated by the adaptor proteins DAP12 and DAP10, as TREM2 itself lacks an intrinsic signaling domain [50, 51] (Fig. 1). Ligand binding to the extracellular domain of TREM2 initiates downstream signaling cascade. TREM2 ligands include Aβ and a diverse array of lipids, such as phospholipids and sphingolipids. Additionally, TREM2 interacts with apolipoproteins, including apolipoprotein E (ApoE) and apolipoprotein J (ApoJ), as well as lipoproteins like low-density lipoprotein (LDL) and high-density lipoprotein (HDL) [47–49, 52–55]. Upon ligand binding to TREM2, the immunoreceptor tyrosine-based activation motif (ITAM) in DAP12 is phosphorylated by Src family kinases, creating a docking site for spleen tyrosine kinase (SYK). SYK is then recruited and activated, initiating downstream signaling [10]. Concurrently, DAP10, through its cytosolic YXNM motif, directly recruits the PI3K subunit to activate the PI3K pathway, thereby amplifying the cellular response to TREM2 activation [10]. SYK and PI3K activation subsequently trigger multiple downstream pathways that regulate diverse cellular processes (Fig. 1). Key signaling cascades downstream of SYK include phospholipase C gamma 2 (PLCγ2), Rac1/Cdc42, ERK and PI3K [51, 56, 57]. These pathways regulate distinct yet interconnected microglial functions. Specifically, PLCγ2 activation is critical for microglial phagocytosis and lipid metabolism [56]. Meanwhile, Rac1/Cdc42-GTPase signaling plays a key role in cytoskeletal remodeling and cell migration [57]. Additionally, the ERK pathway is essential for microglial survival, proliferation, and inflammatory responses [51]. In parallel, the PI3K/Akt pathway, activated by both DAP10 and SYK, governs essential cellular processes such as survival, proliferation, and glucose metabolism [11, 58–60]. Collectively, these pathways coordinate microglial responses to environmental cues, highlighting the central role of TREM2 signaling in microglial functions.

Beyond its role in TREM2 signaling, DAP12 itself has emerged as a key regulator of microglial functions in AD [61]. Genetic studies have identified heterozygous loss-of-function variants in TYROBP, the gene encoding DAP12, which are strongly associated with AD risk [30]. This underscores the importance of intact DAP12-dependent pathways in disease modulation. While the precise functional consequences of altered DAP12 signaling remain an area of active investigation, it is evident that defects in this adaptor protein profoundly impact microglial responses to pathological insults.

The TREM2-DAP12 signaling pathway is dynamically regulated. ​As demonstrated in osteoclasts, phosphorylation of DAP12 following receptor cross-linking recruits SH2-containing inositol polyphosphate phosphatase 1 (SHIP1), which antagonizes SYK activation and dampens downstream signaling [51]. ​While this mechanism has not been directly validated in microglia, the conservation of DAP12-SHIP1-SYK signaling modules across myeloid lineages suggests potential functional relevance. This recruitment serves as a negative regulatory mechanism, fine-tuning TREM2-dependent activation. ​A ligand avidity-dependent model extrapolated from these findings proposes that low-avidity ligands preferentially drive SHIP1 recruitment to suppress signaling, whereas high-avidity ligands that induce TREM2 cross-linking promote robust SYK-dependent activation. ​This hypothesis could explain why heterozygous TREM2 variants impairing ligand binding mimic low-affinity ligand effects, shifting the balance toward inhibition.

In conclusion, TREM2 orchestrates multiple microglial functions, including cell survival, proliferation, migration, phagocytosis and metabolism, through a complex intracellular signaling network. Understanding how genetic variants affect TREM2 structure and function is critical for elucidating the mechanisms underlying neurodegenerative diseases and may provide valuable insights for potential therapeutic strategies.

### TREM2 and glucose metabolism

AD is characterized by regional brain hypometabolism, a hallmark dysfunction measurable using 2-Deoxy-2-[18 F]fluoro-D-glucose positron emission tomography (FDG-PET) [62]. Large-scale proteomic analyses of brain and CSF samples from AD patients reveal significant alterations in glucose metabolism, which correlate with both AD pathology and cognitive decline [63]. Key glycolytic genes, such as pyruvate kinase muscle isoform (PKM) and lactate dehydrogenase B (LDHB), are upregulated in AD, highlighting the central role of glucose metabolism in disease progression [64]. Among various cell types, microglia play a particularly important role in AD-related metabolic changes [63].

Acute exposure to Aβ induces metabolic reprogramming in microglia, shifting energy production from oxidative phosphorylation to glycolysis [65]. This shift is critical for initiating inflammatory responses and involves increased glucose uptake, enhanced lactate production, and upregulation of glycolytic enzymes [64, 66]. Interestingly, lactate production further amplifies glycolysis through histone lactylation, which upregulates glycolytic gene expression in a positive feedback loop [64]. Disrupting this feedback loop has been shown to restore microglial protective functions, although how Aβ induces metabolic pathway alterations in microglia remains incompletely understood.

The metabolic fitness of microglia is markedly impaired in TREM2 knockout (KO) models [58]. TREM2, through its adaptors DAP12 and DAP10, activates the mechanistic target of rapamycin (mTOR) signaling pathway, which plays a crucial role in regulating metabolic pathways and protein synthesis [11, 58]. Loss of TREM2 impairs mTOR activation, leading to reduced ATP production and biosynthesis. In vivo FDG-PET imaging of TREM2 KO and TREM2 T66M knock-in mice shows a significant reduction in cerebral glucose metabolism [67, 68]. This decrease may correlate with impaired glucose uptake by microglia. Supporting this, ex vivo measurements of isolated microglia from TREM2 KO animals reveal lower FDG uptake [68].

Given the pivotal role of microglial metabolism in AD, targeting this process represents a promising therapeutic strategy. Agents such as interferon-γ (IFN-γ) and cyclocreatine, which enhance ATP production, have been shown to restore microglial functions and mitigate AD pathology [58, 65]. Notably, TREM2-activating antibodies boost microglial energy metabolism by promoting mitochondrial fatty acid and glucose oxidation [69]. Moreover, translocator protein (TSPO)-PET and FDG-PET imaging have demonstrated that TREM2 activation enhances microglial activity and glucose metabolism in amyloid mouse models. Thus, targeting TREM2 and microglial metabolism may complement existing AD therapies, which primarily focus on amyloid clearance and synaptic dysfunction, providing a more comprehensive approach to disease intervention.

### TREM2 and lipid metabolism

Lipid metabolism is crucial for maintaining microglial functions and CNS homeostasis, influencing cellular membrane integrity, energy storage, and inflammatory responses. Emerging evidence identifies TREM2 as a key regulator of lipid metabolism in the brain. TREM2 binds a diverse range of lipids, including anionic and zwitterionic species such as sphingomyelin, phosphatidic acid, phosphatidylinositol, phosphatidylcholine, phosphatidylglycerol, phosphatidylserine (PtdSer) and sulfatide [49, 53, 70]. Among these, PtdSer is the most abundant negatively charged phospholipid in the inner leaflet of the plasma membrane in eukaryotic cells [71]. In neurodegenerative conditions, PtdSer externalization on damaged or apoptotic neurons serves as an “eat-me” signal, triggering TREM2-dependent microglial synaptic pruning and cell clearance [72]. Super-resolution microscopy and in vivo imaging studies have demonstrated that Aβ oligomer-induced hyperactive synapses expose PtdSer, marking them for TREM2-mediated engulfment, which helps mitigate neuronal hyperactivity in AD models. Additionally, individuals carrying TREM2 loss-of-function variants exhibit an accumulation of apoptotic-like synapses [72], underscoring TREM2’s essential role in synaptic homeostasis during early AD pathology. Beyond synaptic pruning, TREM2 facilitates the recognition and clearance of damaged cells. Notably, over-expression of TREM2 in non-phagocytic cells, such as Chinese hamster ovary (CHO) and HEK293 cells, enables them to engulf apoptotic neurons, highlighting TREM2’s function in lipid sensing and phagocytosis [16, 73]. This broad lipid-binding capability underscores TREM2’s critical role in modulating microglial responses to neurodegenerative insults and preserving neuronal health.

TREM2 also regulates microglial responses during demyelination, as observed in aging, cuprizone treatment, and lysolecithin injection models. In wild-type (WT) animals, microglial clusters, termed nodules, form in the white matter and increase with age [74]. These nodules are involved in clearing degenerated myelin, and their formation depends on TREM2, as fewer clusters are observed in TREM2 KO or loss-of-function mutant animals [67, 74]. In aged TREM2-deficient animals, myelin debris accumulation correlates with reduced myelin integrity, increased axonal damage, and fewer mature oligodendrocytes in the striatum, underscoring TREM2’s protective role in aging [75]. In the cuprizone model of toxic demyelination, TREM2’s involvement in microglial activation is further confirmed. After cuprizone treatment, microglia exhibit an amoeboid-activated morphology [76]. However, in TREM2 KO mice, microglia maintain a more ramified morphology with less microgliosis. At the transcriptomic level, microglia from TREM2-deficient animals fail to upregulate genes related to activation, phagocytosis, and lipid metabolism, impairing lysosomal and phagosomal function, disrupting oxidative phosphorylation, and altering cholesterol metabolism [76–78]. This failure to clear myelin debris leads to impaired axonal transport, axonal dystrophies, and worsened locomotor deficits in TREM2 KO mice compared to WT controls. Similarly, in the lysolecithin-induced demyelination model, TREM2-deficient mice show a marked accumulation of myelin debris and impaired lesion recovery. This is evidenced by a significant reduction in the number of remyelinated axons, as observed via transmission electron microscopy 3 to 8 weeks post-injection [79, 80]. These findings suggest that TREM2 deficiency impairs myelin clearance, likely due to defects in phagocytosis, reduced lysosomal degradation, or a combination of both.

Another key aspect of TREM2 function is its regulation of lipid droplet formation. In the lysolecithin injection model, TREM2 deficiency led to a significant reduction in lipid droplets within microglia and macrophages at lesion sites, as indicated by immunostaining for perilipin 2, a key structural component of lipid droplets [79]. The impaired biogenesis of lipid droplets and cholesterol esterification in TREM2-deficient microglia was associated with elevated endoplasmic reticulum stress, ultimately hindering the remyelination process. In contrast, in the cuprizone model, TREM2 deficiency resulted in excessive accumulation of cholesteryl ester in microglia [77]. This accumulation is associated with elevated plasma neurofilament light chain (Nf-L) levels, a marker of axonal damage, indicating a link between TREM2 deficiency, lipid dysregulation, and neuronal injury. Further supporting this connection, human induced pluripotent stem cell (iPSC)-derived microglia-like cells (iMGLs) with TREM2 KO exhibit lipid accumulation following myelin treatment, as demonstrated by Nile Red staining [56]. This accumulation is dependent on downstream PLCγ2 activity. These findings highlight the critical role of TREM2 in lipid metabolism. The variations in lipid droplet accumulation observed across different models may stem from differences in experimental design—such as the method of demyelination (local demyelination induced by lysolecithin injection versus global demyelination triggered by cuprizone feeding), the time points analyzed (e.g., a 0.2% cuprizone diet for 5 or 12 weeks during demyelination versus 21 and 62 days post-lysolecithin injection during remyelination versus iMGLs exposed to myelin debris for 48 h), or the lipid visualization techniques employed (perilipin 2 staining versus Nile Red staining). Despite these discrepancies, these studies collectively reinforce the protective role of TREM2 in remyelination and neuronal function. However, the precise molecular mechanisms by which TREM2 regulates lipid metabolism and the downstream events leading to altered lipid levels in its absence are not fully understood. Addressing these gaps through future research will be essential for unraveling the complex relationship between TREM2 signaling, lipid metabolism, and neurodegeneration.

### TREM2 and amyloid pathology

**Fig. 2:**
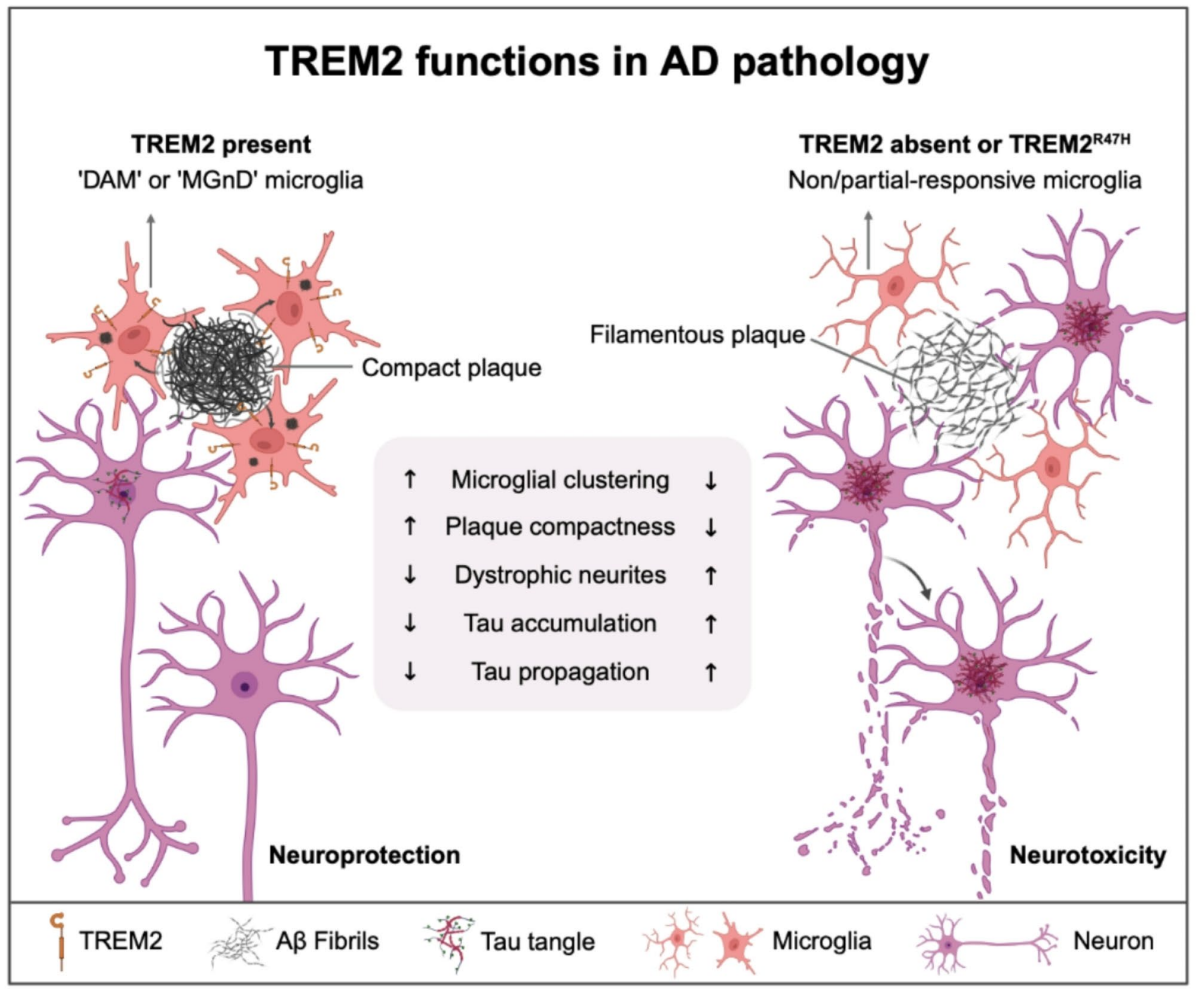
Roles of TREM2 in Alzheimer’s disease pathology. In the presence of TREM2, amyloid plaque accumulation activates the disease-associated microglia (DAM) program and/or the microglial neurodegenerative (MGnD) phenotype. Microglia cluster around plaques, trimming the peripheral region of amyloid-β fibrils and compacting the plaque structure. Additionally, they form a protective barrier between plaques and surrounding neural tissue, reducing amyloid-β-induced neuritic dystrophy. In contrast, TREM2 deficiency or mutation disrupts the activation of DAM and/or the MGnD phenotype, significantly decreasing the number of plaque-associated microglia. This results in the formation of loosely packed amyloid-β plaques termed filamentous plaque, accompanied by more severe neuritic dystrophy in adjacent areas. Consequently, tau pathology becomes more pronounced, promoting its accumulation and propagation, which ultimately accelerates neuronal loss

Aβ accumulation is one of the hallmark pathological features of AD, contributing to neuroinflammation, synaptic dysfunction, and neuronal loss. TREM2 plays a central role in mediating microglial responses to Aβ, thus serving as a key player in the brain’s defense against Aβ pathology. TREM2 directly binds to Aβ, exhibiting particularly high affinity for Aβ42 oligomers due to the irreversibility of this interaction, whereas its binding to Aβ42 and Aβ40 monomers is much weaker and reversible [47, 48, 81]. The binding of Aβ to TREM2 triggers a cascade of microglial responses that involve activation, clustering, and enhanced clearance of these toxic aggregates in a TREM2-dependent manner [47, 48, 81, 82] (Fig. 2). Moreover, TREM2 facilitates the clustering of microglia around Aβ plaques, an essential component of the microglial response to amyloid deposition [82–84]. This clustering helps form a protective barrier around Aβ plaques, sequestering the toxic Aβ and modulating local inflammation, thereby limiting its neurotoxic effects [85]. In the absence of TREM2, microglial clustering is significantly impaired, leading to more diffuse and neuritic plaque morphology, which exacerbates the neurotoxic effects of Aβ [82, 83, 85, 86] (Fig. 2). Studies in TREM2 KO PS2APP mice have shown that the absence of TREM2 leads to an increase in the Aβ42:Aβ40 ratio and the accumulation of soluble, fibrillar Aβ oligomers [87]. Despite a reduction in plaque load at later ages, these alterations are linked to exacerbated axonal dystrophy, dendritic spine loss, and elevated levels of Nf-L in the CSF [87]. Notably, the more diffuse plaque morphology observed in TREM2-deficient mice, along with the altered Aβ profile, highlights the critical neuroprotective role of TREM2 in regulating Aβ aggregation and mitigating neuronal damage. These impairments in microglial responses to Aβ are not restricted to animal models; similar observations have been made in human AD patients carrying TREM2 mutations, where reduced microglial clustering around Aβ plaques and increased neuronal dystrophy are evident, reinforcing the vital role of TREM2 in protecting against Aβ toxicity [82, 85].

TREM2 plays a pivotal role in modulating microglial responses to Aβ accumulation by orchestrating their transition from a homeostatic state to a fully activated disease-associated microglia (DAM) state [88–90]. In amyloidosis mouse models, microglial activation follows a two-step process: an initial TREM2-independent phase characterized by the downregulation of homeostatic checkpoint genes and the upregulation of neurodegeneration-associated markers, followed by a TREM2-dependent phase essential for the induction of lipid metabolism and phagocytosis-related genes involved in Aβ clearance. In the absence of TREM2, microglia become trapped in an intermediate state, failing to fully activate the DAM program or mount an effective response to Aβ accumulation [88–90]. Studies in amyloid mouse models have demonstrated that the loss of functional TREM2 impairs microglial clustering around amyloid plaques, reducing microglial phagocytic capacity and leading to inefficient amyloid clearance [49, 83]. Moreover, TREM2 deficiency results in plaques that are less compact and more fibrillar, which correlates with increased dystrophic neurites and neuronal damage, suggesting that TREM2 plays a critical role in buffering Aβ toxicity [83, 91, 92].

The protective role of TREM2 is particularly evident during the early stages of amyloid deposition. At this stage, TREM2-mediated signaling enhances microglial activation and promotes the clearance of amyloid seeds, thereby limiting plaque formation [82, 93]. Preclinical studies suggest that therapeutic strategies aimed at upregulating TREM2—either through increased gene dosage or activating antibodies—can enhance protective microglial functions and reduce amyloid pathology [93–97]. However, in later stages of amyloid pathology, TREM2 upregulation appears to have little or no impact on plaque burden, suggesting that its therapeutic window may be restricted to early disease progression [93]. These findings underscore the necessity for stage-specific therapeutic strategies that take into account the dynamic role of TREM2 in AD progression. Overall, TREM2 is widely regarded as protective in amyloid pathology by promoting microglial clustering, phagocytosis, and plaque compaction while reducing neurotoxicity. However, its precise effects may be influenced by factors such as disease stage, genetic background, and environmental conditions. Future research should aim to refine our understanding of these variables and develop targeted therapeutic approaches that maximize the beneficial effects of TREM2 while minimizing potential risks.

### TREM2 and tau pathology

Tau protein aggregation and hyperphosphorylation are central to the formation of neurofibrillary tangles, a hallmark of AD and other tauopathies. These tau aggregates disrupt neuronal function and connectivity, contributing significantly to neurodegeneration. The role of TREM2 in tauopathies is complex and context-dependent, with its effects often influenced by the presence of Aβ pathology. Studies using mouse models with concurrent Aβ and tau pathologies, such as the TauPS2APP model, reveal that TREM2 deficiency exacerbates tau accumulation and propagation, particularly in regions associated with Aβ plaques [98]. This suggests that TREM2-dependent modulation of microglial responses is crucial for limiting Aβ-induced tau spreading. Specifically, in the context of Aβ plaques, TREM2 deficiency promotes tau seeding in dystrophic neurites surrounding plaques [99, 100]. This heightened tau seeding correlates with reduced microglial clustering around plaques and elevated levels of neurotoxic Aβ42, reinforcing the idea that TREM2-mediated microglial responses help constrain tau propagation by mitigating Aβ toxicity and protecting surrounding neurons [98–100] (Fig. 2).

In contrast, TREM2’s role in tauopathy models without amyloid pathology, for example the PS19 and hTau models, is less well-defined, with studies producing mixed results. In the hTau mouse model, characterized by human MAPT overexpression, TREM2 deficiency accelerates tau phosphorylation and aggregation during early disease progression, although its impact on neurodegeneration and brain atrophy has not been fully assessed [101]. Conversely, in the PS19 model, which expresses the human tau T34 isoform (one N-terminal insert and four microtubule binding repeats, 1N4R) with the P301S mutation, complete TREM2 knockout reduces brain atrophy associated with tau pathology [102]. This protective effect of TREM2 loss is linked to decreased microgliosis and neuroinflammation, suggesting that TREM2-driven microglial activation may exacerbate neurodegeneration in certain tauopathies. Supporting this, studies have shown that complete TREM2 loss mitigates tau pathology and brain atrophy in the PS19 model [103]. However, heterozygous TREM2 deficiency shows the opposite effect, intensifying tau pathology, brain atrophy, and pro-inflammatory responses in the same model [103]. These contrasting findings highlight the complex effects of TREM2 on tau pathology and the need for caution when extrapolating mouse model results to humans. While TREM2 deletion causes fatal neurodegenerative diseases in humans, such as NHD, TREM2 knockout mice generally exhibit normal phenotypes and do not develop similar conditions. This discrepancy suggests that species-specific differences may influence TREM2’s involvement in tau pathology and neurodegeneration. Further studies are required to elucidate these differences and clarify TREM2’s role in human tauopathies.

The TREM2 R47H variant offers additional insights into TREM2’s role in the PS19 tauopathy model, although findings remain inconsistent. In female PS19 mice, where the R47H variant was introduced via CRISPR/Cas9-mediated knock-in of human TREM2 R47H cDNA replacing endogenous mTrem2, spatial learning and memory deficits were observed compared to mice expressing the common human TREM2 variant, despite no significant differences in tau pathology [104]. Furthermore, these R47H-expressing mice exhibited exacerbated pro-inflammatory responses, suggesting that the R47H variant alters microglial functions in a manner that worsens neuroinflammation and cognitive decline, independent of tau burden. In contrast, in PS19 models where the R47H variant was introduced via bacterial artificial chromosome technology, investigators observed reduced tau pathology, attenuated synaptic loss, and decreased brain atrophy, accompanied by diminished inflammatory responses [105]. These discrepancies are likely driven by differences in the disease models and may also reflect sex-dependent effects, underscoring the complexity of studying TREM2 R47H in tauopathies. Importantly, studies have shown that the TREM2 R47H variant disrupts splicing and decreases Trem2 mRNA and protein levels in mice, but not in humans [106]. As a result, generating humanized TREM2 R47H knock-in mice would be crucial to better understand the cellular effects of the human TREM2 R47H coding variant.

The interaction between TREM2 and ApoE4 introduces an additional layer of complexity in tauopathy. In PS19 tauopathy mice, the presence of the TREM2 R47H variant exacerbates brain atrophy specifically in 9- to 10-month-old female APOE4 mice, while male mice remain unaffected [107]. Notably, this ventricular enlargement is absent in female APOE3-TREM2 R47H tauopathy mice, suggesting that APOE isoform differences influence the impact of TREM2 R47H on neurodegeneration. The exacerbated brain atrophy is further accompanied by increased tau hyperphosphorylation in the frontal cortex, indicating a potential mechanistic link between TREM2 dysfunction, ApoE4, and tau pathology. Mechanistically, this phenotype is associated with an amplified microglial response, characterized by heightened activation of the cyclic GMP-AMP synthase (cGAS)-stimulator of interferon genes (STING) pathway and its downstream interferon response. This dysregulated signaling cascade may contribute to microglial senescence, further accelerating tau pathology and neurodegeneration. Supporting this idea, in PS19 mice expressing APOE4, complete Trem2 knockout exacerbates neurodegeneration and tau pathology, suggesting that TREM2-mediated microglial functions may exert a protective role in the presence of APOE4 [108]. This finding contrasts with earlier studies where Trem2 knockout in PS19 mice expressing mouse Apoe led to a reduction in tau pathology and neurodegeneration [102, 103], highlighting a context-dependent interplay between TREM2 and APOE isoforms in tauopathies.

While large-scale genome-wide association studies exploring the co-occurrence of APOE4 and TREM2 R47H alleles in AD patients remain limited, existing clinical data suggest a functional interaction between these variants. Individuals carrying both APOE4 and TREM2 R47H exhibit a shorter disease duration compared to APOE4 carriers without TREM2 R47H [109]. Additionally, one study reported that AD symptoms are more prevalent in individuals harboring both APOE4 and TREM2 R47H than in those carrying TREM2 R47H with APOE3, further suggesting that APOE4 may exacerbate TREM2 R47H-associated vulnerability [110]. However, a separate large-scale analysis, including neuropathologically confirmed AD cases, found that a subset of individuals carrying only the TREM2 R47H variant—without APOE4—also develop AD pathology [111], indicating that TREM2 R47H alone may be sufficient to drive disease progression in some cases.

In summary, the evidence strongly supports a protective role for TREM2 in amyloid-induced tauopathy, a model more closely resembling AD pathology. However, TREM2’s role in tauopathy without amyloid pathology remains ambiguous and complex, with outcomes varying based on disease context and models. Further research is needed to fully elucidate how TREM2 modulates tau pathology, particularly in the presence of different genetic backgrounds and environmental factors.

## TREM2 as a soluble form (sTREM2)

### The biogenesis of sTREM2

**Fig. 3:**
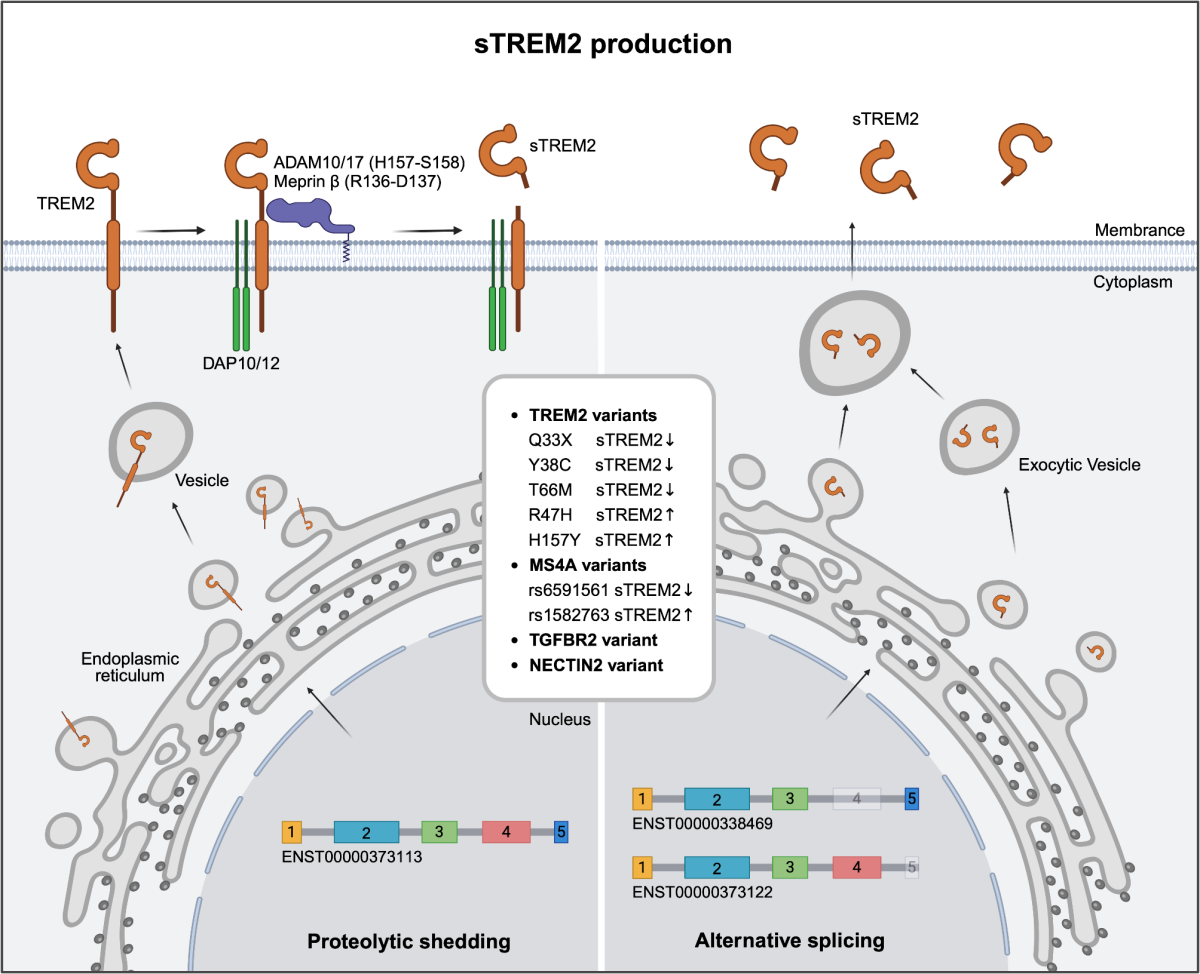
Schematic illustration of sTREM2 production in microglia. This diagram illustrates two primary mechanisms underlying the generation of sTREM2: the proteolytic cleavage pathway and the splicing pathway. In the proteolytic cleavage pathway, the ectodomain of TREM2 is cleaved by metalloproteases, with ADAM10/17 cleaving at the H157-S158 bond and meprin β cleaving at the R136-D137 bond, resulting in the release of sTREM2 into the extracellular space. Alternatively, in the splicing pathway, mRNA variants of TREM2 are generated through splicing events, producing soluble forms of TREM2 that are also released extracellularly. Specifically, ENST00000373113 represents the canonical TREM2 transcript, consisting of five exons, while ENST00000338469 lacks exon 4, which encodes the transmembrane domain. Additionally, ENST00000373122 lacks exon 5 and has an alternative start site at exon 4, resulting in a different coding sequence. The box in the middle highlights the genetic modifiers influencing sTREM2 levels in the CSF

The biogenesis of sTREM2 is a multifaceted process involving two primary pathways: proteolytic shedding and alternative splicing (Fig. 3). Proteolytic shedding of TREM2 is primarily mediated by enzymes of a disintegrin and metalloprotease (ADAM) family, with ADAM10 and ADAM17 being the key players in this process [15–17, 112, 113]. These enzymes cleave the extracellular domain of TREM2 at the histidine 157–serine 158 (H157-S158) site, as confirmed by mass spectrometry analysis [15, 112, 113]. While ADAM17 has been implicated in sTREM2 production in human macrophages and CHO cell lines [15], other studies emphasize the role of ADAM10 in TREM2 shedding in HEK293 cells and murine microglia [16, 112, 113]. The exact roles of ADAM10, ADAM17, and other ADAM family members in sTREM2 generation are not yet fully understood, underscoring the need for further investigation into their individual contributions. In addition to ADAM proteases, meprin β, a zinc metalloproteinase from the astacin family, has been identified as an alternative sheddase [114]. It cleaves TREM2 at a distinct arginine 136–aspartate 137 (R136-D137) site, contributing to sTREM2 production particularly in macrophages. This highlights the diversity of proteolytic pathways involved in TREM2 shedding.

Alongside proteolytic shedding, sTREM2 can also be generated via alternative splicing of TREM2 mRNA (Fig. 3). This process produces several transcript variants, some of which lack the transmembrane domain, leading to the generation of soluble isoforms. In the human brain, four major TREM2 transcripts have been identified: ENST00000373113 (canonical transcript), ENST00000373122 (missing exon 5 and has an alternative start site at exon 4), ENST00000338469 (lacking exon 4), and TREM2Δe2 (missing exon 2) [14, 115]. Among these, ENST00000338469, which lacks the transmembrane domain, represents approximately 20–25% of the total TREM2 mRNA in the brain [14]. Notably, both the ENST00000338469 and ENST00000373122 isoforms are translated and secreted as sTREM2, further supporting the idea that alternative splicing plays a significant role in sTREM2 production [116].

In summary, sTREM2 is generated through two complementary mechanisms: (i) proteolytic shedding of the TREM2 ectodomain and (ii) translation of alternatively spliced TREM2 transcripts lacking the transmembrane domain. While both processes contribute to sTREM2 production, the precise balance between these pathways and their individual roles in different cell types and conditions remain areas for further exploration. A more thorough understanding of these mechanisms is essential for elucidating the biological significance of sTREM2, particularly in the context of neurodegenerative diseases such as AD.

### Genetic modifiers of sTREM2 levels

CSF levels of sTREM2 are notably elevated in neurodegenerative disorders, positioning sTREM2 as a dynamic biomarker that reflects key pathological processes in AD. Consequently, understanding the genetic factors that influence sTREM2 levels has emerged as a critical area of research, providing critical insights into their contributions to AD pathogenesis and potential avenues for therapeutic intervention. A major focus has been on variants within the TREM2 gene itself (Fig. 3). NHD and FTD-associated TREM2 variants, including Q33X, Y38C, and T66M, are associated with significantly reduced CSF sTREM2 levels compared to non-carriers [16, 117]. These reductions align with studies showing that such variants impair the cell-surface localization of TREM2, thereby decreasing its proteolytic shedding into sTREM2 [16]. In contrast, the AD-associated R47H variant is linked to elevated CSF sTREM2 levels, suggesting enhanced shedding or altered regulation [117, 118]. Similarly, the H157Y variant accelerates TREM2 shedding, further highlighting the diverse effects of TREM2 variants on sTREM2 dynamics [112, 119]. Collectively, these findings highlight the complex interplay between TREM2 genetic variants and the regulation of sTREM2 production, underscoring the importance of further research to better understand these mechanisms and their implications for neurodegenerative diseases.

Beyond variants in TREM2, other genetic loci have been identified as modifiers of sTREM2 levels, with the membrane-spanning 4-domains subfamily A (MS4A) gene cluster being particularly significant. MS4A proteins are transmembrane receptors involved in cell activation by working as ion channels or by modulating the signaling of other immunoreceptors. They play key roles in different pathological settings, including cancer, infectious diseases, and neurodegeneration [120]. Variants such as rs1582763 in the MS4A region are associated with increased CSF sTREM2 levels, reduced AD risk, and delayed disease onset [121]. Conversely, the rs6591561 variant, encoding the MS4A4A p.M159V protein, is linked to lower CSF sTREM2 levels, heightened AD risk, and earlier disease onset. Single-nucleus transcriptomic analysis of brain tissue from carriers of these variants identified a distinct microglial subpopulation regulated by MS4A4A expression [122]. The protective variant enhances MS4A4A expression, shifting microglia toward an anti-inflammatory state characterized by the expression of interferon and lipid metabolism genes. In contrast, the risk variant in MS4A4A suppresses this cell state, promoting pro-inflammatory cytokine pathways and impairing lipid metabolism. These findings highlight a mechanistic link between MS4A4A regulation, microglial functions, and sTREM2 generation, presenting potential therapeutic avenues targeting the MS4A pathway to modulate microglial resilience and AD progression.

Other novel genetic modifiers of sTREM2 levels include loci associated with the Transforming Growth Factor Beta Receptor 2 (TGFBR2) and Nectin Cell Adhesion Molecule 2 (NECTIN2) genes [123]. TGFBR2 is a receptor for TGF-β signaling, which regulates various cellular processes including cell growth, differentiation, immune response, and fibrosis [124]. NECTIN2 is a cell adhesion molecule critical for forming synaptic junctions, immune synapses, and viral entry [125]. Its genetic associations have been implicated in a variety of human diseases, including AD, coronary heart disease, and multiple sclerosis. Variants such as rs73823326 in the TGFBR2 region and rs11666329 in the NECTIN2 region have been linked to altered CSF sTREM2 levels [123]. Lentivirus-mediated overexpression of NECTIN2 or TGFBR2 in macrophages derived from human peripheral blood mononuclear cells results in a significant increase in extracellular sTREM2 levels, without affecting total TREM2 expression. This suggests the role of NECTIN2 and TGFBR2 in selectively modulating sTREM2 levels. Both genes are highly expressed in microglia and encode transmembrane proteins, suggesting they may regulate CSF sTREM2 by influencing the proteolytic cleavage of TREM2 at the cell membrane. These findings emphasize the potential of NECTIN2 and TGFBR2 as novel therapeutic targets for AD, highlighting the importance of understanding genetic factors that influence sTREM2 levels in neurodegeneration.

### Dynamic changes of sTREM2 levels across AD progression

**Fig. 4:**
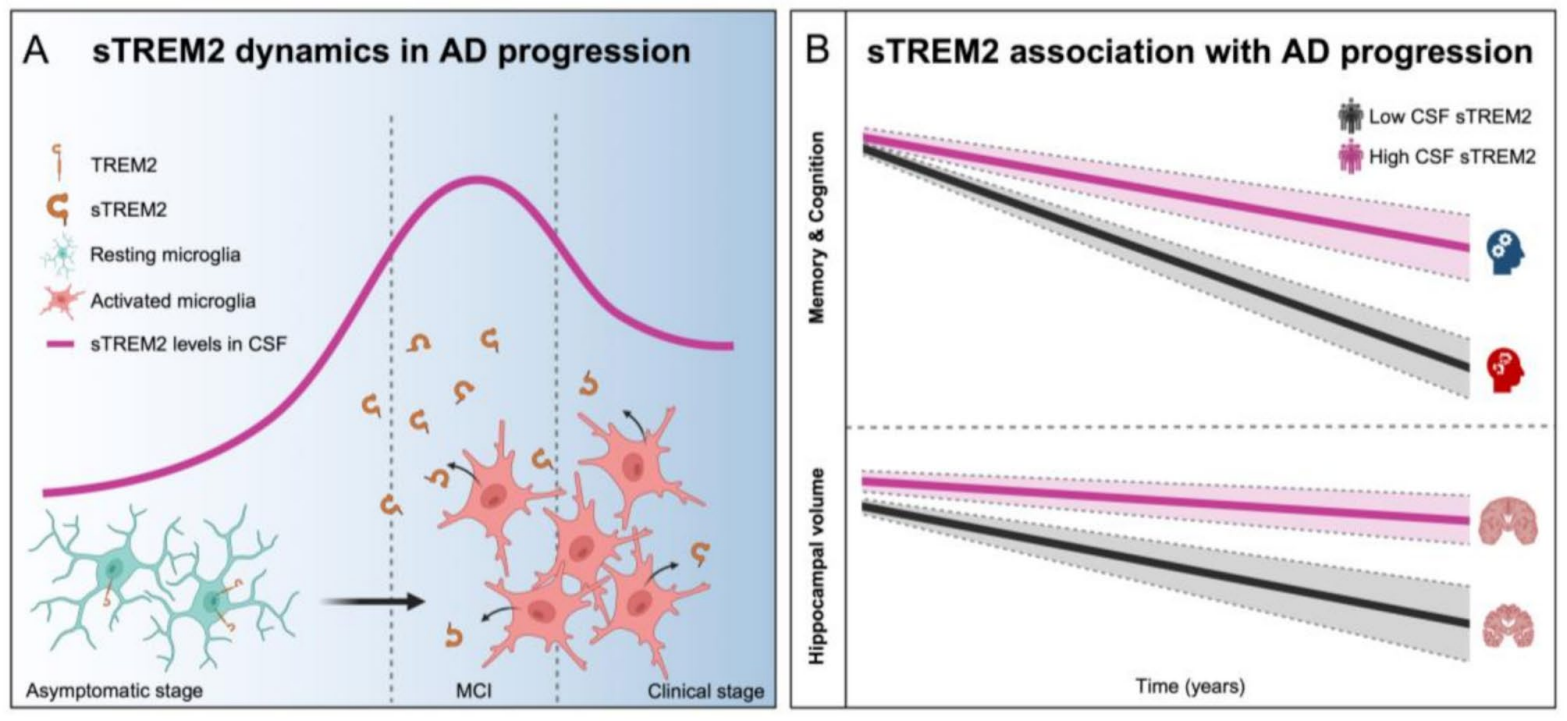
Dynamic changes of sTREM2 across AD spectrum. (**A**) The levels of sTREM2 in CSF gradually increase throughout the AD continuum, correlating with microglial activation. This increase reaches its peak during the mild cognitive impairment (MCI) stage and subsequently plateaus or even declines in the dementia stage. (**B**) In elderly individuals, higher baseline CSF sTREM2 concentrations are associated with slower cognitive decline, particularly in memory and overall cognition. Furthermore, elevated sTREM2 levels correlate with a slower rate of hippocampal atrophy

The role of sTREM2 as a biomarker in AD has attracted significant interest due to its capacity to reflect neuroinflammatory processes and track the stages of disease progression. Cross-sectional studies consistently show that CSF sTREM2 levels are elevated in AD patients compared to cognitively normal individuals, supporting its relevance in AD pathology [18, 19, 21, 117, 126]. Notably, sTREM2 levels exhibit stage-specific variation, peaking during the early symptomatic stages of AD (Fig. 4A). Data from the Dominantly Inherited Alzheimer Network (DIAN) reveals that sTREM2 levels increase in familial AD mutation carriers up to five years before and after symptom onset [19], highlighting its potential as an early biomarker for neuroinflammatory changes that may precede clinical manifestations.

The relationship between elevated CSF sTREM2 levels, cognitive function, and AD pathology has been well documented. Studies indicate that in mild cognitive impairment (MCI) patients, higher sTREM2 levels correlate with increased gray matter volume in brain regions vulnerable to AD [127]. Longitudinal research further shows that higher baseline sTREM2 levels are associated with slower cognitive decline over an 11-year period in AD patients who test positive for key AD biomarkers, such as Aβ42 and p-tau181 [21] (Fig. 4B). Additionally, a higher sTREM2 to p-tau181 ratio predicts a slower rate of progression from MCI to AD dementia. In autosomal dominant AD, elevated sTREM2 levels have been linked to reduced Aβ pathology and slower cognitive decline, particularly during the pre-symptomatic phase, further emphasizing sTREM2’s potential neuroprotective role [23]. Interestingly, higher CSF sTREM2 levels also appear to mitigate the ApoE4-associated risk of cognitive decline and AD-related neurodegeneration, suggesting that sTREM2 may have broader implications in neurodegenerative disease risk [128].

While these findings highlight the importance of sTREM2 as a biomarker in AD, it remains unclear whether the elevated sTREM2 levels reflect an overall increase in TREM2 expression due to inflammation, selective enhancement of proteolytic processing, or altered splicing of TREM2. The precise mechanisms contributing to the dynamics of sTREM2 in AD pathogenesis warrant further exploration. Elevated sTREM2 levels may reflect increased proteolytic cleavage of TREM2 at the cell membrane, potentially regulated by factors like MS4A4A, TGFBR2 or NECTIN2, which can selectively modulate sTREM2 shedding without affecting total TREM2 expression. Alternatively, changes in TREM2 splicing could also contribute to higher sTREM2 levels, although this remains to be investigated in more detail. Understanding these mechanisms will provide deeper insight into the role of sTREM2 in AD and its potential for therapeutic targeting.

Taken together, these findings underscore the complex and dynamic role of sTREM2 in AD progression, positioning it as both a valuable biomarker and a potential therapeutic target. Ongoing longitudinal studies are critical to fully elucidate the mechanisms through which sTREM2 influences disease progression and to explore its application in therapeutic strategies aimed at modifying AD’s trajectory.

### sTREM2 in amyloid and tau pathology

**Fig. 5:**
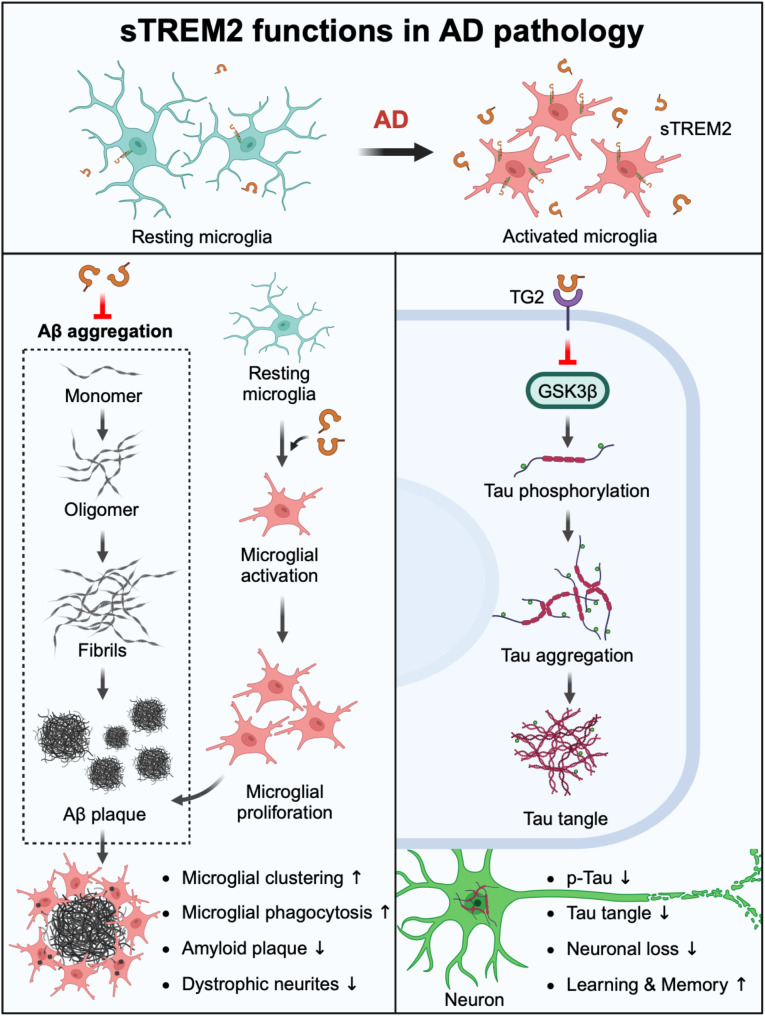
Potential roles of sTREM2 in Alzheimer’s disease pathology. The sTREM2, generated upon microglial activation, plays a crucial role in modulating AD pathology. It has been shown to inhibit Aβ oligomerization, fibrillization, and neurotoxicity. Additionally, sTREM2 promotes microglial activation, enhancing several microglial functions, including increased cell proliferation, migration, clustering around amyloid plaques, and the uptake and degradation of Aβ. These actions collectively reduce amyloid plaque burden and the number of dystrophic neurites. Additionally, sTREM2 interacts with transgelin-2 (TG2), leading to the deactivation of GSK3β and reducing tau phosphorylation. This process helps prevent neuronal loss and alleviates cognitive and behavioral impairments

sTREM2 plays a critical role in neurodegenerative diseases, especially AD, by influencing both amyloid and tau pathologies (Fig. 5). Research suggests that sTREM2 functions with chaperone-like activity, helping mitigate the toxic effects of Aβ [129]. In vitro studies have shown that sTREM2 preferentially binds to oligomeric forms of Aβ, inhibiting their aggregation and reducing the neurotoxic effects that accelerate disease progression. While these findings suggest that sTREM2 may help limit the damaging consequences of Aβ accumulation, further in vivo studies are needed to confirm the relevance of this mechanism in AD brain. Conversely, the R47H variant of sTREM2 exhibits reduced binding affinity for Aβ oligomers, which could promote harmful Aβ aggregation and increase toxicity.

In addition to its role in modulating Aβ aggregation, sTREM2 plays a pivotal role in regulating key microglial functions, including survival, proliferation, migration, and phagocytosis. These processes are essential for maintaining brain homeostasis and mounting an effective response to pathological changes in neurodegenerative diseases such as AD. Our study in primary microglial cultures demonstrated that sTREM2 enhances microglial survival through a PI3K/Akt-dependent pathway and stimulates the production of inflammatory cytokines via NF-κB signaling [130]. Importantly, sTREM2 induces microglial activation in the mouse brain independently of full-length TREM2. Furthermore, in the 5xFAD mouse model, sTREM2 promotes microglial proliferation, facilitates migration and clustering around amyloid plaques, and enhances the uptake and degradation of Aβ [20]. However, the R47H variant significantly impairs sTREM2’s ability to promote microglial survival and activate immune responses [130], which may contribute to a more aggressive disease course in AD patients carrying this variant.

Additional evidence supporting the protective role of sTREM2 in AD comes from in vivo experiments using transgenic AD mouse models. Administration of recombinant sTREM2 to amyloidogenic mice has been shown to promote microglial clustering around amyloid plaques, which may reduce neurite dystrophy associated with these deposits [20] (Fig. 5). Moreover, sTREM2 reduces amyloid plaque load and improves cognitive function, highlighting its potential as a therapeutic agent for preventing or reversing amyloid pathology [20]. Interestingly, studies indicate that specific fragments of sTREM2 are more efficient at binding Aβ and reducing amyloid deposition compared to full-length sTREM2, suggesting that these selected fragments could serve as more effective therapeutic interventions [131]. Gene therapy approaches involving adeno-associated virus (AAV)-mediated overexpression of sTREM2 have conferred significant neuroprotection, improving brain function and reducing amyloid pathology in transgenic mice [20]. In contrast, studies using transgenic mice with altered TREM2 shedding—via mutations in the ADAM protease cleavage site—reveal that reduced membrane TREM2 cleavage exacerbates amyloid deposition and neuronal degeneration, highlighting the importance of regulated TREM2 shedding for optimal brain health [132].

In addition to its role in amyloid pathology, sTREM2 has also been implicated in tau pathophysiology (Fig. 5). Elevated CSF levels of sTREM2 are associated with slower tau aggregation in Aβ-positive individuals who do not yet exhibit dementia, suggesting that sTREM2 may delay tau-related neurodegeneration in the preclinical stages of AD [133]. Functional magnetic resonance imaging (MRI) analyses further support these findings, linking higher CSF sTREM2 levels to reduced tau deposition across key brain regions involved in AD. These data underscore sTREM2’s potential role in the early stages of tau pathology and its promise as a biomarker for tau-related changes in AD. In PS19 mice, AAV-mediated overexpression of sTREM2 significantly attenuates tau pathology, preventing tau aggregation and rescuing cognitive and behavioral deficits (Fig. 5). This reinforces the idea that increasing sTREM2 expression may offer therapeutic benefits against tau-related neurodegeneration [22]. Additionally, sTREM2 has been shown to interact with transgelin-2 (TG2), deactivates the GSK3β and ameliorates tau phosphorylation. These findings suggest that sTREM2 plays a role in stabilizing tau, thereby reducing its neurotoxic effects.

In summary, these findings highlight the dual protective roles of sTREM2 in both amyloid and tau pathologies in AD. By modulating immune responses and mitigating the neurotoxic aggregation of pathological proteins, sTREM2 emerges as a promising therapeutic target. As research advances, sTREM2 may play a pivotal role in the development of novel interventions aimed at slowing or even reversing the progression of AD. This positions sTREM2 not only as a biomarker for early disease detection but also as a potential therapeutic target for a range of neurodegenerative disorders. Continued exploration of the specific molecular mechanisms by which sTREM2 regulates AD pathology will be crucial for developing therapeutic strategies that promote neuroprotection in AD.

## Future perspectives and clinical implications

TREM2, both as a cell-surface receptor and in its soluble form, has emerged as a pivotal player in AD and other neurodegenerative disorders. Partial loss-of-function variants of TREM2 are strongly associated with increased AD risk, underscoring its protective role in maintaining microglial homeostasis. Concurrently, elevated CSF levels of sTREM2 have been correlated with slower cognitive decline and attenuated disease progression, further emphasizing its clinical significance. These findings collectively highlight TREM2 and sTREM2 as promising targets for therapeutic intervention. However, the precise mechanisms through which they modulate microglial functions and influence amyloid and tau pathologies remain incompletely understood, warranting further research. Future studies should focus on delineating the molecular mechanisms that regulate TREM2 and sTREM2 and exploring their interplay with genetic and environmental factors.

Therapeutic strategies targeting TREM2 and sTREM2 represent complementary approaches with significant potential in mitigating AD. Agonistic antibodies designed to activate TREM2 signaling have shown preclinical efficacy by enhancing microglial protective functions, reducing amyloid burden, and mitigating neuroinflammation [13, 69, 96, 97, 134, 135]. These promising findings have led to ongoing clinical trials evaluating the safety and therapeutic benefits of TREM2 activation in AD patients [13]. Despite these promising findings, the therapeutic modulation of TREM2 remains complex. While studies on TREM2 deficiency suggest that TREM2 constrains amyloid-induced tau pathology, chronic treatment with the agonistic TREM2 antibody AL002a has been reported to enhance the seeding and spreading of phosphorylated tau, exacerbating neuritic dystrophy [136]. These findings underscore the context-dependent nature of TREM2-targeting therapies, highlighting the need for further investigations to determine the optimal timing, disease stage, and microglial functional states that shape treatment outcomes.

Meanwhile, the neuroprotective properties of sTREM2 position it as an additional target for therapeutic development. Both preclinical and clinical evidence linking elevated sTREM2 levels to reduced amyloid and tau pathologies, as well as improved cognitive outcomes, underscores the rationale for interventions aimed at enhancing sTREM2 production or mimicking its protective effects. A combination of TREM2-targeted therapies and sTREM2-based interventions could potentially provide synergistic benefits for comprehensive disease modification. However, it is important to note that several TREM2 agonistic antibodies reportedly bind to the same epitope shared by full-length TREM2 and sTREM2 [69, 95]. This interaction may interfere with the protective functions of sTREM2, warranting careful consideration in the design of therapeutic strategies.

The timing and context of these interventions are critical, as the roles of TREM2 and sTREM2 appear to be dependent on the disease stage. Long-term therapeutic strategies must take into account these stage-specific roles to maximize their efficacy. For example, during the early stages of amyloid seeding, TREM2 signaling plays a crucial role in facilitating the clearance of amyloid seeds, thereby preventing further plaque formation [82, 93]. However, its effects appear to be less pronounced in later stages of amyloid pathology. Similarly, sTREM2 levels fluctuate in a stage-dependent manner, peaking during the early symptomatic phase of AD [18, 19]. Elevated sTREM2 levels have been associated with a reduction in Aβ accumulation and a slower rate of cognitive decline, particularly in the pre-symptomatic stage [21, 23]. These findings underscore the stage-specific roles of TREM2 and sTREM2 in AD pathology, highlighting the importance of precise therapeutic timing and the use of biomarkers that reflect disease progression and microglial activity to optimize intervention strategies.

From a diagnostic perspective, sTREM2 shows considerable potential as a biomarker for the early detection of AD and for monitoring disease progression. Studies using TSPO-PET imaging have demonstrated a correlation between sTREM2 levels in CSF and TSPO signaling in neurodegenerative diseases [69, 137], indicating that sTREM2 may serve as a potential marker for microglial activation. Since microglial activation is an early and pivotal event in the pathogenesis of AD, CSF sTREM2 could be a valuable marker for monitoring this process in clinical settings. Moreover, sTREM2 levels can help distinguish AD patients at various stages of the disease, facilitating more tailored and personalized treatment strategies. Monitoring sTREM2 levels during treatment may enable clinicians to assess the effectiveness of a therapeutic intervention and make timely adjustments as needed. Additionally, elevated CSF sTREM2 levels have been suggested to be associated with a decreased risk of MCI-to-AD conversion [138]. Therefore, CSF sTREM2 levels may serve as a predictor of cognitive outcomes, enabling the identification of patients who are more likely to benefit from specific interventions and enhancing the precision of clinical decision-making.

In summary, TREM2 and sTREM2 represent interconnected therapeutic and diagnostic opportunities in AD. Advancing our understanding of their roles, regulatory mechanisms, and clinical applications will enable the development of targeted interventions that can modify disease progression. Integrating these insights into precision medicine strategies holds substantial potential to enhance the quality of life for patients with AD and related neurodegenerative disorders.

## Data Availability

Not applicable.

## Abbreviations

Aβ: Amyloid-β
AAV: Adeno-associated virus
AD: Alzheimer's disease
ADAM: A disintegrin and metalloprotease
ApoE: Apolipoprotein E
ApoJ: Apolipoprotein J
cGAS: Cyclic GMP-AMP synthase
CHO: Chinese hamster ovary
CNS: Central nervous system
CSF: Cerebrospinal fluid
DAM: Disease-associated microglia
DAP12: DNAX-activating protein 12
DIAN: Dominantly inherited alzheimer network
FDG-PET: Fluoro-D-glucose positron emission tomography
FTD: Frontotemporal dementia
HDL: High-density lipoprotein
IFN-γ: Interferon-γ
iMGLs: Human iPSC-derived microglia-like cells
iPSC: Induced pluripotent stem cell
ITAM: Immunoreceptor tyrosine-based activation motif
KO: Knockout
LDHB: Lactate dehydrogenase B
LDL: Low-density lipoprotein
MCI: Mild cognitive impairment
MRI: Magnetic resonance imaging
MS4A: Membrane-spanning 4-domains subfamily A
mTOR: Mechanistic target of rapamycin
NECTIN2: Nectin cell adhesion molecule 2
Nf-L: Neurofilament light chain
NHD: Nasu-Hakola disease
Ors: Odds ratios
PKM: Pyruvate kinase muscle isoform
PLCγ2: Phospholipase C gamma 2
PtdSer: Phosphatidylserine
SHIP1: SH2-containing inositol polyphosphate phosphatase 1
STING: Stimulator of interferon genes
sTREM2: Soluble TREM2
SYK: Spleen tyrosine kinase
TG2: Transgelin-2
TGFBR2: Transforming growth factor beta receptor 2
TREM2: Triggering receptor expressed on myeloid cells 2
TSPO: Translocator protein
WT: Wild-type

## References

[1] Braak H, Braak E. Neuropathological stageing of Alzheimer-related changes. Acta Neuropathol. 1991;82:239–59. 1759558 10.1007/BF00308809

[2] Hardy JA, Higgins GA. Alzheimer’s disease: the amyloid cascade hypothesis. Science. 1992;256:184–5. 1566067 10.1126/science.1566067

[3] Heneka MT, Carson MJ, El Khoury J, Landreth GE, Brosseron F, Feinstein DL, Jacobs AH, Wyss-Coray T, Vitorica J, Ransohoff RM, et al. Neuroinflammation in Alzheimer’s disease. Lancet Neurol. 2015;14:388–405. 25792098 10.1016/S1474-4422(15)70016-5 PMC5909703

[4] Colonna M, Butovsky O. Microglia function in the central nervous system during health and neurodegeneration. Annu Rev Immunol. 2017;35:441–68. 28226226 10.1146/annurev-immunol-051116-052358 PMC8167938

[5] Sarlus H, Heneka MT. Microglia in Alzheimer’s disease. J Clin Invest. 2017;127:3240–9. 28862638 10.1172/JCI90606 PMC5669553

[6] Guerreiro R, Wojtas A, Bras J, Carrasquillo M, Rogaeva E, Majounie E, Cruchaga C, Sassi C, Kauwe JS, Younkin S, et al. TREM2 variants in Alzheimer’s disease. N Engl J Med. 2013;368:117–27. 23150934 10.1056/NEJMoa1211851 PMC3631573

[7] Jonsson T, Stefansson H, Steinberg S, Jonsdottir I, Jonsson PV, Snaedal J, Bjornsson S, Huttenlocher J, Levey AI, Lah JJ, et al. Variant of TREM2 associated with the risk of Alzheimer’s disease. N Engl J Med. 2013;368:107–16. 23150908 10.1056/NEJMoa1211103 PMC3677583

[8] Deczkowska A, Weiner A, Amit I. The physiology, pathology, and potential therapeutic applications of the TREM2 signaling pathway. Cell. 2020;181:1207–17. 32531244 10.1016/j.cell.2020.05.003

[9] Jay TR, von Saucken VE, Landreth GE. TREM2 in neurodegenerative diseases. Mol Neurodegener. 2017;12:56. 28768545 10.1186/s13024-017-0197-5 PMC5541421

[10] Colonna M. The biology of TREM receptors. Nat Rev Immunol. 2023;23:580–94. 36750615 10.1038/s41577-023-00837-1 PMC9904274

[11] Wang S, Sudan R, Peng V, Zhou Y, Du S, Yuede CM, Lei T, Hou J, Cai Z, Cella M, et al. TREM2 drives microglia response to amyloid-beta via SYK-dependent and -independent pathways. Cell. 2022;185:4153–e41694119. 36306735 10.1016/j.cell.2022.09.033 PMC9625082

[12] Kober DL, Brett TJ. TREM2-ligand interactions in health and disease. J Mol Biol. 2017;429:1607–29. 28432014 10.1016/j.jmb.2017.04.004 PMC5485854

[13] Schlepckow K, Morenas-Rodriguez E, Hong S, Haass C. Stimulation of TREM2 with agonistic antibodies-an emerging therapeutic option for Alzheimer’s disease. Lancet Neurol. 2023;22:1048–60. 37863592 10.1016/S1474-4422(23)00247-8

[14] Del-Aguila JL, Benitez BA, Li Z, Dube U, Mihindukulasuriya KA, Budde JP, Farias FHG, Fernandez MV, Ibanez L, Jiang S, et al. TREM2 brain transcript-specific studies in AD and TREM2 mutation carriers. Mol Neurodegener. 2019;14:18. 31068200 10.1186/s13024-019-0319-3 PMC6505298

[15] Feuerbach D, Schindler P, Barske C, Joller S, Beng-Louka E, Worringer KA, Kommineni S, Kaykas A, Ho DJ, Ye C, et al. ADAM17 is the main Sheddase for the generation of human triggering receptor expressed in myeloid cells (hTREM2) ectodomain and cleaves TREM2 after histidine 157. Neurosci Lett. 2017;660:109–14. 28923481 10.1016/j.neulet.2017.09.034

[16] Kleinberger G, Yamanishi Y, Suarez-Calvet M, Czirr E, Lohmann E, Cuyvers E, Struyfs H, Pettkus N, Wenninger-Weinzierl A, Mazaheri F, et al. TREM2 mutations implicated in neurodegeneration impair cell surface transport and phagocytosis. Sci Transl Med. 2014;6:243ra286. 10.1126/scitranslmed.3009093 24990881

[17] Wunderlich P, Glebov K, Kemmerling N, Tien NT, Neumann H, Walter J. Sequential proteolytic processing of the triggering receptor expressed on myeloid cells-2 (TREM2) protein by ectodomain shedding and gamma-secretase-dependent intramembranous cleavage. J Biol Chem. 2013;288:33027–36. 24078628 10.1074/jbc.M113.517540 PMC3829152

[18] Suarez-Calvet M, Kleinberger G, Araque Caballero MA, Brendel M, Rominger A, Alcolea D, Fortea J, Lleo A, Blesa R, Gispert JD, et al. sTREM2 cerebrospinal fluid levels are a potential biomarker for microglia activity in early-stage Alzheimer’s disease and associate with neuronal injury markers. EMBO Mol Med. 2016;8:466–76. 26941262 10.15252/emmm.201506123 PMC5120370

[19] Suarez-Calvet M, Araque Caballero MA, Kleinberger G, Bateman RJ, Fagan AM, Morris JC, Levin J, Danek A, Ewers M, Haass C. Dominantly inherited alzheimer N: early changes in CSF sTREM2 in dominantly inherited alzheimer’s disease occur after amyloid deposition and neuronal injury. Sci Transl Med. 2016;8:369ra178. 27974666 10.1126/scitranslmed.aag1767 PMC5385711

[20] Zhong L, Xu Y, Zhuo R, Wang T, Wang K, Huang R, Wang D, Gao Y, Zhu Y, Sheng X, et al. Soluble TREM2 ameliorates pathological phenotypes by modulating microglial functions in an Alzheimer’s disease model. Nat Commun. 2019;10:1365. 30911003 10.1038/s41467-019-09118-9 PMC6433910

[21] Ewers M, Franzmeier N, Suarez-Calvet M, Morenas-Rodriguez E, Caballero MAA, Kleinberger G, Piccio L, Cruchaga C, Deming Y, Dichgans M et al. Increased soluble TREM2 in cerebrospinal fluid is associated with reduced cognitive and clinical decline in Alzheimer’s disease. Sci Transl Med. 2019;11. 10.1126/scitranslmed.aav6221 PMC7050285 31462511

[22] Zhang X, Tang L, Yang J, Meng L, Chen J, Zhou L, Wang J, Xiong M, Zhang Z. Soluble TREM2 ameliorates tau phosphorylation and cognitive deficits through activating transgelin-2 in Alzheimer’s disease. Nat Commun. 2023;14:6670. 37865646 10.1038/s41467-023-42505-x PMC10590452

[23] Morenas-Rodriguez E, Li Y, Nuscher B, Franzmeier N, Xiong C, Suarez-Calvet M, Fagan AM, Schultz S, Gordon BA, Benzinger TLS, et al. Soluble TREM2 in CSF and its association with other biomarkers and cognition in autosomal-dominant Alzheimer’s disease: a longitudinal observational study. Lancet Neurol. 2022;21:329–41. 35305339 10.1016/S1474-4422(22)00027-8 PMC8926925

[24] Guerreiro RJ, Lohmann E, Bras JM, Gibbs JR, Rohrer JD, Gurunlian N, Dursun B, Bilgic B, Hanagasi H, Gurvit H, et al. Using exome sequencing to reveal mutations in TREM2 presenting as a frontotemporal dementia-like syndrome without bone involvement. JAMA Neurol. 2013;70:78–84. 23318515 10.1001/jamaneurol.2013.579 PMC4001789

[25] Paloneva J, Mandelin J, Kiialainen A, Bohling T, Prudlo J, Hakola P, Haltia M, Konttinen YT, Peltonen L. DAP12/TREM2 deficiency results in impaired osteoclast differentiation and osteoporotic features. J Exp Med. 2003;198:669–75. 12925681 10.1084/jem.20030027 PMC2194176

[26] Paloneva J, Manninen T, Christman G, Hovanes K, Mandelin J, Adolfsson R, Bianchin M, Bird T, Miranda R, Salmaggi A, et al. Mutations in two genes encoding different subunits of a receptor signaling complex result in an identical disease phenotype. Am J Hum Genet. 2002;71:656–62. 12080485 10.1086/342259 PMC379202

[27] Soragna D, Papi L, Ratti MT, Sestini R, Tupler R, Montalbetti L. An Italian family affected by Nasu-Hakola disease with a novel genetic mutation in the TREM2 gene. J Neurol Neurosurg Psychiatry. 2003;74:825–6. 12754369 10.1136/jnnp.74.6.825-a PMC1738498

[28] Dardiotis E, Siokas V, Pantazi E, Dardioti M, Rikos D, Xiromerisiou G, Markou A, Papadimitriou D, Speletas M, Hadjigeorgiou GM. A novel mutation in TREM2 gene causing Nasu-Hakola disease and review of the literature. Neurobiol Aging. 2017;53:194. e113-194 e122. 10.1016/j.neurobiolaging.2017.01.015 28214109

[29] Klunemann HH, Ridha BH, Magy L, Wherrett JR, Hemelsoet DM, Keen RW, De Bleecker JL, Rossor MN, Marienhagen J, Klein HE, et al. The genetic causes of basal ganglia calcification, dementia, and bone cysts: DAP12 and TREM2. Neurology. 2005;64:1502–7. 15883308 10.1212/01.WNL.0000160304.00003.CA

[30] Stefansson H, Walters GB, Sveinbjornsson G, Tragante V, Einarsson G, Helgason H, Sigurethsson A, Beyter D, Snaebjarnarson AS, Ivarsdottir EV, et al. Homozygosity for R47H in TREM2 and the risk of Alzheimer’s disease. N Engl J Med. 2024;390:2217–9. 38899702 10.1056/NEJMc2314334

[31] Finelli D, Rollinson S, Harris J, Jones M, Richardson A, Gerhard A, Snowden J, Mann D, Pickering-Brown S. TREM2 analysis and increased risk of Alzheimer’s disease. Neurobiol Aging. 2015;36:e546549–513. 10.1016/j.neurobiolaging.2014.08.001 25260849

[32] Ghani M, Sato C, Kakhki EG, Gibbs JR, Traynor B, St George-Hyslop P, Rogaeva E. Mutation analysis of the MS4A and TREM gene clusters in a case-control Alzheimer’s disease data set. Neurobiol Aging. 2016;42:217. e217-217 e213. 10.1016/j.neurobiolaging.2016.03.009 PMC8985522 27084067

[33] Hooli BV, Parrado AR, Mullin K, Yip WK, Liu T, Roehr JT, Qiao D, Jessen F, Peters O, Becker T, et al. The rare TREM2 R47H variant exerts only a modest effect on alzheimer disease risk. Neurology. 2014;83:1353–8. 25186855 10.1212/WNL.0000000000000855 PMC4189101

[34] Jin SC, Benitez BA, Karch CM, Cooper B, Skorupa T, Carrell D, Norton JB, Hsu S, Harari O, Cai Y, et al. Coding variants in TREM2 increase risk for Alzheimer’s disease. Hum Mol Genet. 2014;23:5838–46. 24899047 10.1093/hmg/ddu277 PMC4189899

[35] Sims R, van der Lee SJ, Naj AC, Bellenguez C, Badarinarayan N, Jakobsdottir J, Kunkle BW, Boland A, Raybould R, Bis JC, et al. Rare coding variants in PLCG2, ABI3, and TREM2 implicate microglial-mediated innate immunity in Alzheimer’s disease. Nat Genet. 2017;49:1373–84. 28714976 10.1038/ng.3916 PMC5669039

[36] Slattery CF, Beck JA, Harper L, Adamson G, Abdi Z, Uphill J, Campbell T, Druyeh R, Mahoney CJ, Rohrer JD, et al. R47H TREM2 variant increases risk of typical early-onset Alzheimer’s disease but not of prion or frontotemporal dementia. Alzheimers Dement. 2014;10:602–e608604. 25160042 10.1016/j.jalz.2014.05.1751 PMC4627504

[37] Jin SC, Carrasquillo MM, Benitez BA, Skorupa T, Carrell D, Patel D, Lincoln S, Krishnan S, Kachadoorian M, Reitz C, et al. TREM2 is associated with increased risk for Alzheimer’s disease in African Americans. Mol Neurodegener. 2015;10:19. 25886450 10.1186/s13024-015-0016-9 PMC4426167

[38] Jiao B, Liu X, Tang B, Hou L, Zhou L, Zhang F, Zhou Y, Guo J, Yan X, Shen L. Investigation of TREM2, PLD3, and UNC5C variants in patients with Alzheimer’s disease from Mainland China. Neurobiol Aging. 2014;35:2422–e2429. 10.1016/j.neurobiolaging.2014.04.025 24866402

[39] Ma J, Zhou Y, Xu J, Liu X, Wang Y, Deng Y, Wang G, Xu W, Ren R, Liu X, et al. Association study of TREM2 polymorphism rs75932628 with late-onset Alzheimer’s disease in Chinese Han population. Neurol Res. 2014;36:894–6. 24725293 10.1179/1743132814Y.0000000376

[40] Wang P, Guo Q, Zhou Y, Chen K, Xu Y, Ding D, Hong Z, Zhao Q. Lack of association between triggering receptor expressed on myeloid cells 2 polymorphism rs75932628 and late-onset Alzheimer’s disease in a Chinese Han population. Psychiatr Genet. 2018;28:16–8. 29256968 10.1097/YPG.0000000000000188 PMC5757673

[41] Yu JT, Jiang T, Wang YL, Wang HF, Zhang W, Hu N, Tan L, Sun L, Tan MS, Zhu XC, Tan L. Triggering receptor expressed on myeloid cells 2 variant is rare in late-onset Alzheimer’s disease in Han Chinese individuals. Neurobiol Aging. 2014;35:e937931–933. 10.1016/j.neurobiolaging.2013.10.075 24184202

[42] Miyashita A, Wen Y, Kitamura N, Matsubara E, Kawarabayashi T, Shoji M, Tomita N, Furukawa K, Arai H, Asada T, et al. Lack of genetic association between TREM2 and late-onset Alzheimer’s disease in a Japanese population. J Alzheimers Dis. 2014;41:1031–8. 24762945 10.3233/JAD-140225

[43] Rayaprolu S, Mullen B, Baker M, Lynch T, Finger E, Seeley WW, Hatanpaa KJ, Lomen-Hoerth C, Kertesz A, Bigio EH, et al. TREM2 in neurodegeneration: evidence for association of the p.R47H variant with frontotemporal dementia and Parkinson’s disease. Mol Neurodegener. 2013;8:19. 23800361 10.1186/1750-1326-8-19 PMC3691612

[44] Jiang T, Tan L, Chen Q, Tan MS, Zhou JS, Zhu XC, Lu H, Wang HF, Zhang YD, Yu JT. A rare coding variant in TREM2 increases risk for Alzheimer’s disease in Han Chinese. Neurobiol Aging. 2016;42:e217211–213. 10.1016/j.neurobiolaging.2016.02.023 27067662

[45] Song W, Hooli B, Mullin K, Jin SC, Cella M, Ulland TK, Wang Y, Tanzi RE, Colonna M. Alzheimer’s disease-associated TREM2 variants exhibit either decreased or increased ligand-dependent activation. Alzheimers Dement. 2017;13:381–7. 27520774 10.1016/j.jalz.2016.07.004 PMC5299056

[46] Kober DL, Alexander-Brett JM, Karch CM, Cruchaga C, Colonna M, Holtzman MJ, Brett TJ. Neurodegenerative disease mutations in TREM2 reveal a functional surface and distinct loss-of-function mechanisms. Elife. 2016;5. 10.7554/eLife.20391 PMC5173322 27995897

[47] Zhao Y, Wu X, Li X, Jiang LL, Gui X, Liu Y, Sun Y, Zhu B, Pina-Crespo JC, Zhang M, et al. TREM2 is a receptor for beta-amyloid that mediates microglial function. Neuron. 2018;97:1023–e10311027. 29518356 10.1016/j.neuron.2018.01.031 PMC5889092

[48] Zhong L, Wang Z, Wang D, Wang Z, Martens YA, Wu L, Xu Y, Wang K, Li J, Huang R, et al. Amyloid-beta modulates microglial responses by binding to the triggering receptor expressed on myeloid cells 2 (TREM2). Mol Neurodegener. 2018;13:15. 29587871 10.1186/s13024-018-0247-7 PMC5870375

[49] Wang Y, Cella M, Mallinson K, Ulrich JD, Young KL, Robinette ML, Gilfillan S, Krishnan GM, Sudhakar S, Zinselmeyer BH, et al. TREM2 lipid sensing sustains the microglial response in an Alzheimer’s disease model. Cell. 2015;160:1061–71. 25728668 10.1016/j.cell.2015.01.049 PMC4477963

[50] Daws MR, Lanier LL, Seaman WE, Ryan JC. Cloning and characterization of a novel mouse myeloid DAP12-associated receptor family. Eur J Immunol. 2001;31:783–91. 11241283 10.1002/1521-4141(200103)31:3<783::aid-immu783>3.0.co;2-u

[51] Peng Q, Malhotra S, Torchia JA, Kerr WG, Coggeshall KM, Humphrey MB. TREM2- and DAP12-dependent activation of PI3K requires DAP10 and is inhibited by SHIP1. Sci Signal. 2010;3:ra38. 20484116 10.1126/scisignal.2000500 PMC2900152

[52] Atagi Y, Liu CC, Painter MM, Chen XF, Verbeeck C, Zheng H, Li X, Rademakers R, Kang SS, Xu H, et al. Apolipoprotein E is a ligand for triggering receptor expressed on myeloid cells 2 (TREM2). J Biol Chem. 2015;290:26043–50. 26374899 10.1074/jbc.M115.679043 PMC4646257

[53] Cannon JP, O’Driscoll M, Litman GW. Specific lipid recognition is a general feature of CD300 and TREM molecules. Immunogenetics. 2012;64:39–47. 21800138 10.1007/s00251-011-0562-4

[54] Yeh FL, Wang Y, Tom I, Gonzalez LC, Sheng M. TREM2 binds to apolipoproteins, including APOE and CLU/APOJ, and thereby facilitates uptake of amyloid-beta by microglia. Neuron. 2016;91:328–40. 27477018 10.1016/j.neuron.2016.06.015

[55] Bailey CC, DeVaux LB, Farzan M. The triggering receptor expressed on myeloid cells 2 binds Apolipoprotein E. J Biol Chem. 2015;290:26033–42. 26374897 10.1074/jbc.M115.677286 PMC4646256

[56] Andreone BJ, Przybyla L, Llapashtica C, Rana A, Davis SS, van Lengerich B, Lin K, Shi J, Mei Y, Astarita G, et al. Alzheimer’s-associated PLCgamma2 is a signaling node required for both TREM2 function and the inflammatory response in human microglia. Nat Neurosci. 2020;23:927–38. 32514138 10.1038/s41593-020-0650-6

[57] Rong Z, Cheng B, Zhong L, Ye X, Li X, Jia L, Li Y, Shue F, Wang N, Cheng Y, et al. Activation of FAK/Rac1/Cdc42-GTPase signaling ameliorates impaired microglial migration response to Abeta(42) in triggering receptor expressed on myeloid cells 2 loss-of-function murine models. FASEB J. 2020;34:10984–97. 32613609 10.1096/fj.202000550RR

[58] Ulland TK, Song WM, Huang SC, Ulrich JD, Sergushichev A, Beatty WL, Loboda AA, Zhou Y, Cairns NJ, Kambal A, et al. TREM2 maintains microglial metabolic fitness in Alzheimer’s disease. Cell. 2017;170:649–e663613. 28802038 10.1016/j.cell.2017.07.023 PMC5573224

[59] Ennerfelt H, Frost EL, Shapiro DA, Holliday C, Zengeler KE, Voithofer G, Bolte AC, Lammert CR, Kulas JA, Ulland TK, Lukens JR. SYK coordinates neuroprotective microglial responses in neurodegenerative disease. Cell. 2022;185:4135–e41524122. 36257314 10.1016/j.cell.2022.09.030 PMC9617784

[60] Zheng H, Jia L, Liu CC, Rong Z, Zhong L, Yang L, Chen XF, Fryer JD, Wang X, Zhang YW, et al. TREM2 promotes microglial survival by activating Wnt/beta-catenin pathway. J Neurosci. 2017;37:1772–84. 28077724 10.1523/JNEUROSCI.2459-16.2017 PMC5320608

[61] Zhang B, Gaiteri C, Bodea LG, Wang Z, McElwee J, Podtelezhnikov AA, Zhang C, Xie T, Tran L, Dobrin R, et al. Integrated systems approach identifies genetic nodes and networks in late-onset Alzheimer’s disease. Cell. 2013;153:707–20. 23622250 10.1016/j.cell.2013.03.030 PMC3677161

[62] Jack CR Jr., Bennett DA, Blennow K, Carrillo MC, Dunn B, Haeberlein SB, Holtzman DM, Jagust W, Jessen F, Karlawish J, et al. NIA-AA research framework: toward a biological definition of Alzheimer’s disease. Alzheimers Dement. 2018;14:535–62. 29653606 10.1016/j.jalz.2018.02.018 PMC5958625

[63] Johnson ECB, Dammer EB, Duong DM, Ping L, Zhou M, Yin L, Higginbotham LA, Guajardo A, White B, Troncoso JC, et al. Large-scale proteomic analysis of Alzheimer’s disease brain and cerebrospinal fluid reveals early changes in energy metabolism associated with microglia and astrocyte activation. Nat Med. 2020;26:769–80. 32284590 10.1038/s41591-020-0815-6 PMC7405761

[64] Pan RY, He L, Zhang J, Liu X, Liao Y, Gao J, Liao Y, Yan Y, Li Q, Zhou X, et al. Positive feedback regulation of microglial glucose metabolism by histone H4 lysine 12 lactylation in Alzheimer’s disease. Cell Metab. 2022;34:634–e648636. 35303422 10.1016/j.cmet.2022.02.013

[65] Baik SH, Kang S, Lee W, Choi H, Chung S, Kim JI, Mook-Jung I. A breakdown in metabolic reprogramming causes microglia dysfunction in Alzheimer’s disease. Cell Metab. 2019;30:493–e507496. 31257151 10.1016/j.cmet.2019.06.005

[66] Bernier LP, York EM, MacVicar BA. Immunometabolism in the brain: how metabolism shapes microglial function. Trends Neurosci. 2020;43:854–69. 32958333 10.1016/j.tins.2020.08.008

[67] Kleinberger G, Brendel M, Mracsko E, Wefers B, Groeneweg L, Xiang X, Focke C, Deussing M, Suarez-Calvet M, Mazaheri F, et al. The FTD-like syndrome causing TREM2 T66M mutation impairs microglia function, brain perfusion, and glucose metabolism. EMBO J. 2017;36:1837–53. 28559417 10.15252/embj.201796516 PMC5494459

[68] Xiang X, Wind K, Wiedemann T, Blume T, Shi Y, Briel N, Beyer L, Biechele G, Eckenweber F, Zatcepin A, et al. Microglial activation states drive glucose uptake and FDG-PET alterations in neurodegenerative diseases. Sci Transl Med. 2021;13:eabe5640. 34644146 10.1126/scitranslmed.abe5640

[69] van Lengerich B, Zhan L, Xia D, Chan D, Joy D, Park JI, Tatarakis D, Calvert M, Hummel S, Lianoglou S, et al. A TREM2-activating antibody with a blood-brain barrier transport vehicle enhances microglial metabolism in Alzheimer’s disease models. Nat Neurosci. 2023;26:416–29. 36635496 10.1038/s41593-022-01240-0 PMC9991924

[70] Daws MR, Sullam PM, Niemi EC, Chen TT, Tchao NK, Seaman WE. Pattern recognition by TREM-2: binding of anionic ligands. J Immunol. 2003;171:594–9. 12847223 10.4049/jimmunol.171.2.594

[71] Leventis PA, Grinstein S. The distribution and function of phosphatidylserine in cellular membranes. Annu Rev Biophys. 2010;39:407–27. 20192774 10.1146/annurev.biophys.093008.131234

[72] Rueda-Carrasco J, Sokolova D, Lee SE, Childs T, Jurcakova N, Crowley G, De Schepper S, Ge JZ, Lachica JI, Toomey CE, et al. Microglia-synapse engulfment via PtdSer-TREM2 ameliorates neuronal hyperactivity in Alzheimer’s disease models. EMBO J. 2023;42:e113246. 37575021 10.15252/embj.2022113246 PMC10548173

[73] Hsieh CL, Koike M, Spusta SC, Niemi EC, Yenari M, Nakamura MC, Seaman WE. A role for TREM2 ligands in the phagocytosis of apoptotic neuronal cells by microglia. J Neurochem. 2009;109:1144–56. 19302484 10.1111/j.1471-4159.2009.06042.x PMC3087597

[74] Safaiyan S, Besson-Girard S, Kaya T, Cantuti-Castelvetri L, Liu L, Ji H, Schifferer M, Gouna G, Usifo F, Kannaiyan N, et al. White matter aging drives microglial diversity. Neuron. 2021;109:1100–e11171110. 33606969 10.1016/j.neuron.2021.01.027

[75] McCray TJ, Bedford LM, Bissel SJ, Lamb BT. Trem2-deficiency aggravates and accelerates age-related myelin degeneration. Acta Neuropathol Commun. 2024;12:154. 39300502 10.1186/s40478-024-01855-3 PMC11411802

[76] Cantoni C, Bollman B, Licastro D, Xie M, Mikesell R, Schmidt R, Yuede CM, Galimberti D, Olivecrona G, Klein RS, et al. TREM2 regulates microglial cell activation in response to demyelination in vivo. Acta Neuropathol. 2015;129:429–47. 25631124 10.1007/s00401-015-1388-1 PMC4667728

[77] Nugent AA, Lin K, van Lengerich B, Lianoglou S, Przybyla L, Davis SS, Llapashtica C, Wang J, Kim DJ, Xia D, et al. TREM2 regulates microglial cholesterol metabolism upon chronic phagocytic challenge. Neuron. 2020;105:837–e854839. 31902528 10.1016/j.neuron.2019.12.007

[78] Poliani PL, Wang Y, Fontana E, Robinette ML, Yamanishi Y, Gilfillan S, Colonna M. TREM2 sustains microglial expansion during aging and response to demyelination. J Clin Invest. 2015;125:2161–70. 25893602 10.1172/JCI77983 PMC4463196

[79] Gouna G, Klose C, Bosch-Queralt M, Liu L, Gokce O, Schifferer M, Cantuti-Castelvetri L, Simons M. TREM2-dependent lipid droplet biogenesis in phagocytes is required for remyelination. J Exp Med. 2021;218. 10.1084/jem.20210227 PMC8404472 34424266

[80] Wang Y, Kyauk RV, Shen YA, Xie L, Reichelt M, Lin H, Jiang Z, Ngu H, Shen K, Greene JJ, et al. TREM2-dependent microglial function is essential for remyelination and subsequent neuroprotection. Glia. 2023;71:1247–58. 36625077 10.1002/glia.24335

[81] Lessard CB, Malnik SL, Zhou Y, Ladd TB, Cruz PE, Ran Y, Mahan TE, Chakrabaty P, Holtzman DM, Ulrich JD et al. High-affinity interactions and signal transduction between Abeta oligomers and TREM2. EMBO Mol Med. 2018;10. 10.15252/emmm.201809027 PMC6220267 30341064

[82] Parhizkar S, Arzberger T, Brendel M, Kleinberger G, Deussing M, Focke C, Nuscher B, Xiong M, Ghasemigharagoz A, Katzmarski N, et al. Loss of TREM2 function increases amyloid seeding but reduces plaque-associated ApoE. Nat Neurosci. 2019;22:191–204. 30617257 10.1038/s41593-018-0296-9 PMC6417433

[83] Wang Y, Ulland TK, Ulrich JD, Song W, Tzaferis JA, Hole JT, Yuan P, Mahan TE, Shi Y, Gilfillan S, et al. TREM2-mediated early microglial response limits diffusion and toxicity of amyloid plaques. J Exp Med. 2016;213:667–75. 27091843 10.1084/jem.20151948 PMC4854736

[84] Jay TR, Miller CM, Cheng PJ, Graham LC, Bemiller S, Broihier ML, Xu G, Margevicius D, Karlo JC, Sousa GL, et al. TREM2 deficiency eliminates TREM2 + inflammatory macrophages and ameliorates pathology in Alzheimer’s disease mouse models. J Exp Med. 2015;212:287–95. 25732305 10.1084/jem.20142322 PMC4354365

[85] Yuan P, Condello C, Keene CD, Wang Y, Bird TD, Paul SM, Luo W, Colonna M, Baddeley D, Grutzendler J. TREM2 haplodeficiency in mice and humans impairs the microglia barrier function leading to decreased amyloid compaction and severe axonal dystrophy. Neuron. 2016;92:252–64. 27710785 10.1016/j.neuron.2016.09.016

[86] Condello C, Yuan P, Schain A, Grutzendler J. Microglia constitute a barrier that prevents neurotoxic protofibrillar Abeta42 hotspots around plaques. Nat Commun. 2015;6:6176. 25630253 10.1038/ncomms7176 PMC4311408

[87] Meilandt WJ, Ngu H, Gogineni A, Lalehzadeh G, Lee SH, Srinivasan K, Imperio J, Wu T, Weber M, Kruse AJ, et al. Trem2 deletion reduces late-stage amyloid plaque accumulation, elevates the Abeta42:Abeta40 ratio, and exacerbates axonal dystrophy and dendritic spine loss in the PS2APP Alzheimer’s mouse model. J Neurosci. 2020;40:1956–74. 31980586 10.1523/JNEUROSCI.1871-19.2019 PMC7046459

[88] Krasemann S, Madore C, Cialic R, Baufeld C, Calcagno N, El Fatimy R, Beckers L, O’Loughlin E, Xu Y, Fanek Z, et al. The TREM2-APOE pathway drives the transcriptional phenotype of dysfunctional microglia in neurodegenerative diseases. Immunity. 2017;47:566–e581569. 28930663 10.1016/j.immuni.2017.08.008 PMC5719893

[89] Keren-Shaul H, Spinrad A, Weiner A, Matcovitch-Natan O, Dvir-Szternfeld R, Ulland TK, David E, Baruch K, Lara-Astaiso D, Toth B, et al. A unique microglia type associated with restricting development of Alzheimer’s disease. Cell. 2017;169:1276–e12901217. 28602351 10.1016/j.cell.2017.05.018

[90] Zhou Y, Song WM, Andhey PS, Swain A, Levy T, Miller KR, Poliani PL, Cominelli M, Grover S, Gilfillan S, et al. Human and mouse single-nucleus transcriptomics reveal TREM2-dependent and TREM2-independent cellular responses in Alzheimer’s disease. Nat Med. 2020;26:131–42. 31932797 10.1038/s41591-019-0695-9 PMC6980793

[91] Yuan P, Condello C, Keene CD, Wang Y, Bird TD, Paul SM, Luo W, Colonna M, Baddeley D, Grutzendler J. TREM2 haplodeficiency in mice and humans impairs the microglia barrier function leading to decreased amyloid compaction and severe axonal dystrophy. Neuron. 2016;90:724–39. 27196974 10.1016/j.neuron.2016.05.003 PMC4898967

[92] Ulrich JD, Finn MB, Wang Y, Shen A, Mahan TE, Jiang H, Stewart FR, Piccio L, Colonna M, Holtzman DM. Altered microglial response to Abeta plaques in APPPS1-21 mice heterozygous for TREM2. Mol Neurodegener. 2014;9:20. 24893973 10.1186/1750-1326-9-20 PMC4049806

[93] Zhao N, Qiao W, Li F, Ren Y, Zheng J, Martens YA, Wang X, Li L, Liu CC, Chen K et al. Elevating microglia TREM2 reduces amyloid seeding and suppresses disease-associated microglia. J Exp Med. 2022;219. 10.1084/jem.20212479 PMC9481739 36107206

[94] Lee CYD, Daggett A, Gu X, Jiang LL, Langfelder P, Li X, Wang N, Zhao Y, Park CS, Cooper Y, et al. Elevated TREM2 gene dosage reprograms microglia responsivity and ameliorates pathological phenotypes in Alzheimer’s disease models. Neuron. 2018;97:1032–e10481035. 29518357 10.1016/j.neuron.2018.02.002 PMC5927822

[95] Schlepckow K, Monroe KM, Kleinberger G, Cantuti-Castelvetri L, Parhizkar S, Xia D, Willem M, Werner G, Pettkus N, Brunner B, et al. Enhancing protective microglial activities with a dual function TREM2 antibody to the stalk region. EMBO Mol Med. 2020;12:e11227. 32154671 10.15252/emmm.201911227 PMC7136959

[96] Wang S, Mustafa M, Yuede CM, Salazar SV, Kong P, Long H, Ward M, Siddiqui O, Paul R, Gilfillan S et al. Anti-human TREM2 induces microglia proliferation and reduces pathology in an Alzheimer’s disease model. J Exp Med. 2020;217. 10.1084/jem.20200785 PMC7478730 32579671

[97] Zhao P, Xu Y, Jiang L, Fan X, Li L, Li X, Arase H, Zhao Y, Cao W, Zheng H, et al. A tetravalent TREM2 agonistic antibody reduced amyloid pathology in a mouse model of Alzheimer’s disease. Sci Transl Med. 2022;14:eabq0095. 36070367 10.1126/scitranslmed.abq0095

[98] Lee SH, Meilandt WJ, Xie L, Gandham VD, Ngu H, Barck KH, Rezzonico MG, Imperio J, Lalehzadeh G, Huntley MA, et al. Trem2 restrains the enhancement of tau accumulation and neurodegeneration by beta-amyloid pathology. Neuron. 2021;109:1283–e13011286. 33675684 10.1016/j.neuron.2021.02.010

[99] Gratuze M, Chen Y, Parhizkar S, Jain N, Strickland MR, Serrano JR, Colonna M, Ulrich JD, Holtzman DM. Activated microglia mitigate Abeta-associated Tau seeding and spreading. J Exp Med. 2021;218. 10.1084/jem.20210542 PMC8190588 34100905

[100] Leyns CEG, Gratuze M, Narasimhan S, Jain N, Koscal LJ, Jiang H, Manis M, Colonna M, Lee VMY, Ulrich JD, Holtzman DM. TREM2 function impedes tau seeding in neuritic plaques. Nat Neurosci. 2019;22:1217–22. 31235932 10.1038/s41593-019-0433-0 PMC6660358

[101] Bemiller SM, McCray TJ, Allan K, Formica SV, Xu G, Wilson G, Kokiko-Cochran ON, Crish SD, Lasagna-Reeves CA, Ransohoff RM, et al. TREM2 deficiency exacerbates tau pathology through dysregulated kinase signaling in a mouse model of tauopathy. Mol Neurodegener. 2017;12:74. 29037207 10.1186/s13024-017-0216-6 PMC5644120

[102] Leyns CEG, Ulrich JD, Finn MB, Stewart FR, Koscal LJ, Remolina Serrano J, Robinson GO, Anderson E, Colonna M, Holtzman DM. TREM2 deficiency attenuates neuroinflammation and protects against neurodegeneration in a mouse model of tauopathy. Proc Natl Acad Sci U S A. 2017;114:11524–9. 29073081 10.1073/pnas.1710311114 PMC5663386

[103] Sayed FA, Telpoukhovskaia M, Kodama L, Li Y, Zhou Y, Le D, Hauduc A, Ludwig C, Gao F, Clelland C, et al. Differential effects of partial and complete loss of TREM2 on microglial injury response and tauopathy. Proc Natl Acad Sci U S A. 2018;115:10172–7. 30232263 10.1073/pnas.1811411115 PMC6176614

[104] Sayed FA, Kodama L, Fan L, Carling GK, Udeochu JC, Le D, Li Q, Zhou L, Wong MY, Horowitz R, et al. AD-linked R47H-TREM2 mutation induces disease-enhancing microglial states via AKT hyperactivation. Sci Transl Med. 2021;13:eabe3947. 34851693 10.1126/scitranslmed.abe3947 PMC9345574

[105] Gratuze M, Leyns CE, Sauerbeck AD, St-Pierre MK, Xiong M, Kim N, Serrano JR, Tremblay ME, Kummer TT, Colonna M, et al. Impact of TREM2R47H variant on tau pathology-induced gliosis and neurodegeneration. J Clin Invest. 2020;130:4954–68. 32544086 10.1172/JCI138179 PMC7456230

[106] Xiang X, Piers TM, Wefers B, Zhu K, Mallach A, Brunner B, Kleinberger G, Song W, Colonna M, Herms J, et al. The Trem2 R47H Alzheimer’s risk variant impairs splicing and reduces Trem2 mRNA and protein in mice but not in humans. Mol Neurodegener. 2018;13:49. 30185230 10.1186/s13024-018-0280-6 PMC6126019

[107] Carling GK, Fan L, Foxe NR, Norman K, Wong MY, Zhu D, Corona C, Razzoli A, Yu F, Yarahmady A et al. Alzheimer’s disease-linked risk alleles elevate microglial cGAS-associated senescence and neurodegeneration in a tauopathy model. Neuron. 2024;112:3877–96. 10.1016/j.neuron.2024.09.006 PMC11624100 39353433

[108] Gratuze M, Schlachetzki JCM, D’Oliveira Albanus R, Jain N, Novotny B, Brase L, Rodriguez L, Mansel C, Kipnis M, O’Brien S, et al. TREM2-independent microgliosis promotes tau-mediated neurodegeneration in the presence of ApoE4. Neuron. 2023;111:202–e219207. 36368315 10.1016/j.neuron.2022.10.022 PMC9852006

[109] Korvatska O, Leverenz JB, Jayadev S, McMillan P, Kurtz I, Guo X, Rumbaugh M, Matsushita M, Girirajan S, Dorschner MO, et al. R47H variant of TREM2 associated with alzheimer disease in a large late-onset family: clinical, genetic, and neuropathological study. JAMA Neurol. 2015;72:920–7. 26076170 10.1001/jamaneurol.2015.0979 PMC4825672

[110] Murray CE, King A, Troakes C, Hodges A, Lashley T. APOE epsilon4 is also required in TREM2 R47H variant carriers for Alzheimer’s disease to develop. Neuropathol Appl Neurobiol. 2019;45:183–6. 29411406 10.1111/nan.12474

[111] Guerreiro R, Orme T, Naj AC, Kuzma AB, Schellenberg GD, Bras J. Is APOE epsilon4 required for Alzheimer’s disease to develop in TREM2 p.R47H variant carriers? Neuropathol Appl Neurobiol. 2019;45:187–9. 30229991 10.1111/nan.12517 PMC6380937

[112] Schlepckow K, Kleinberger G, Fukumori A, Feederle R, Lichtenthaler SF, Steiner H, Haass C. An Alzheimer-associated TREM2 variant occurs at the ADAM cleavage site and affects shedding and phagocytic function. EMBO Mol Med. 2017;9:1356–65. 28855300 10.15252/emmm.201707672 PMC5623859

[113] Thornton P, Sevalle J, Deery MJ, Fraser G, Zhou Y, Stahl S, Franssen EH, Dodd RB, Qamar S, Gomez Perez-Nievas B, et al. TREM2 shedding by cleavage at the H157-S158 bond is accelerated for the Alzheimer’s disease-associated H157Y variant. EMBO Mol Med. 2017;9:1366–78. 28855301 10.15252/emmm.201707673 PMC5623839

[114] Berner DK, Wessolowski L, Armbrust F, Schneppenheim J, Schlepckow K, Koudelka T, Scharfenberg F, Lucius R, Tholey A, Kleinberger G, et al. Meprin beta cleaves TREM2 and controls its phagocytic activity on macrophages. FASEB J. 2020;34:6675–87. 32237095 10.1096/fj.201902183R

[115] Kiianitsa K, Kurtz I, Beeman N, Matsushita M, Chien WM, Raskind WH, Korvatska O. Novel TREM2 splicing isoform that lacks the V-set immunoglobulin domain is abundant in the human brain. J Leukoc Biol. 2021;110:829–37. 34061398 10.1002/JLB.2HI0720-463RR PMC10433532

[116] Moutinho M, Coronel I, Tsai AP, Di Prisco GV, Pennington T, Atwood BK, Puntambekar SS, Smith DC, Martinez P, Han S, et al. TREM2 splice isoforms generate soluble TREM2 species that disrupt long-term potentiation. Genome Med. 2023;15:11. 36805764 10.1186/s13073-023-01160-z PMC9940368

[117] Piccio L, Deming Y, Del-Aguila JL, Ghezzi L, Holtzman DM, Fagan AM, Fenoglio C, Galimberti D, Borroni B, Cruchaga C. Cerebrospinal fluid soluble TREM2 is higher in alzheimer disease and associated with mutation status. Acta Neuropathol. 2016;131:925–33. 26754641 10.1007/s00401-016-1533-5 PMC4867123

[118] Suarez-Calvet M, Morenas-Rodriguez E, Kleinberger G, Schlepckow K, Araque Caballero MA, Franzmeier N, Capell A, Fellerer K, Nuscher B, Eren E, et al. Early increase of CSF sTREM2 in Alzheimer’s disease is associated with tau related-neurodegeneration but not with amyloid-beta pathology. Mol Neurodegener. 2019;14:1. 30630532 10.1186/s13024-018-0301-5 PMC6327425

[119] Qiao W, Chen Y, Zhong J, Madden BJ, Charlesworth CM, Martens YA, Liu CC, Knight J, Ikezu TC, Kurti A, et al. Trem2 H157Y increases soluble TREM2 production and reduces amyloid pathology. Mol Neurodegener. 2023;18:8. 36721205 10.1186/s13024-023-00599-3 PMC9890893

[120] Mattiola I, Mantovani A, Locati M. The Tetraspan MS4A family in homeostasis, immunity, and disease. Trends Immunol. 2021;42:764–81. 34384709 10.1016/j.it.2021.07.002

[121] Deming Y, Filipello F, Cignarella F, Cantoni C, Hsu S, Mikesell R, Li Z, Del-Aguila JL, Dube U, Farias FG et al. The MS4A gene cluster is a key modulator of soluble TREM2 and Alzheimer’s disease risk. Sci Transl Med. 2019;11. 10.1126/scitranslmed.aau2291 PMC6697053 31413141

[122] You SF, Brase L, Filipello F, Iyer AK, Del-Aguila J, He J, D’Oliveira Albanus R, Budde J, Norton J, Gentsch J et al. MS4A4A modifies the risk of Alzheimer disease by regulating lipid metabolism and immune response in a unique microglia state. medRxiv. 2023.

[123] Wang L, Nykanen NP, Western D, Gorijala P, Timsina J, Li F, Wang Z, Ali M, Yang C, Liu M, et al. Proteo-genomics of soluble TREM2 in cerebrospinal fluid provides novel insights and identifies novel modulators for Alzheimer’s disease. Mol Neurodegener. 2024;19:1. 38172904 10.1186/s13024-023-00687-4 PMC10763080

[124] Vander Ark A, Cao J, Li X. TGF-beta receptors: in and beyond TGF-beta signaling. Cell Signal. 2018;52:112–20. 30184463 10.1016/j.cellsig.2018.09.002

[125] Mizutani K, Miyata M, Shiotani H, Kameyama T, Takai Y. Nectin-2 in general and in the brain. Mol Cell Biochem. 2022;477:167–80. 34633611 10.1007/s11010-021-04241-y

[126] Heslegrave A, Heywood W, Paterson R, Magdalinou N, Svensson J, Johansson P, Ohrfelt A, Blennow K, Hardy J, Schott J, et al. Increased cerebrospinal fluid soluble TREM2 concentration in Alzheimer’s disease. Mol Neurodegener. 2016;11:3. 26754172 10.1186/s13024-016-0071-x PMC4709982

[127] Gispert JD, Suarez-Calvet M, Monte GC, Tucholka A, Falcon C, Rojas S, Rami L, Sanchez-Valle R, Llado A, Kleinberger G, et al. Cerebrospinal fluid sTREM2 levels are associated with gray matter volume increases and reduced diffusivity in early Alzheimer’s disease. Alzheimers Dement. 2016;12:1259–72. 27423963 10.1016/j.jalz.2016.06.005

[128] Franzmeier N, Suarez-Calvet M, Frontzkowski L, Moore A, Hohman TJ, Morenas-Rodriguez E, Nuscher B, Shaw L, Trojanowski JQ, Dichgans M, et al. Higher CSF sTREM2 attenuates ApoE4-related risk for cognitive decline and neurodegeneration. Mol Neurodegener. 2020;15:57. 33032659 10.1186/s13024-020-00407-2 PMC7545547

[129] Vilalta A, Zhou Y, Sevalle J, Griffin JK, Satoh K, Allendorf DH, De S, Puigdellivol M, Bruzas A, Burguillos MA, et al. Wild-type sTREM2 blocks Abeta aggregation and neurotoxicity, but the Alzheimer’s R47H mutant increases Abeta aggregation. J Biol Chem. 2021;296:100631. 33823153 10.1016/j.jbc.2021.100631 PMC8113883

[130] Zhong L, Chen XF, Wang T, Wang Z, Liao C, Wang Z, Huang R, Wang D, Li X, Wu L, et al. Soluble TREM2 induces inflammatory responses and enhances microglial survival. J Exp Med. 2017;214:597–607. 28209725 10.1084/jem.20160844 PMC5339672

[131] Sheng X, Yao Y, Huang R, Xu Y, Zhu Y, Chen L, Zhang L, Wang W, Zhuo R, Can D, et al. Identification of the minimal active soluble TREM2 sequence for modulating microglial phenotypes and amyloid pathology. J Neuroinflammation. 2021;18:286. 34893068 10.1186/s12974-021-02340-7 PMC8665564

[132] Dhandapani R, Neri M, Bernhard M, Brzak I, Schweizer T, Rudin S, Joller S, Berth R, Kernen J, Neuhaus A, et al. Sustained Trem2 stabilization accelerates microglia heterogeneity and Abeta pathology in a mouse model of Alzheimer’s disease. Cell Rep. 2022;39:110883. 35649351 10.1016/j.celrep.2022.110883

[133] Nabizadeh F. sTREM2 is associated with attenuated Tau aggregate accumulation in the presence of amyloid-beta pathology. Brain Commun. 2023;5:fcad286. 37942087 10.1093/braincomms/fcad286 PMC10629471

[134] Fassler M, Rappaport MS, Cuno CB, George J. Engagement of TREM2 by a novel monoclonal antibody induces activation of microglia and improves cognitive function in Alzheimer’s disease models. J Neuroinflammation. 2021;18:19. 33422057 10.1186/s12974-020-01980-5 PMC7796541

[135] Price BR, Sudduth TL, Weekman EM, Johnson S, Hawthorne D, Woolums A, Wilcock DM. Therapeutic Trem2 activation ameliorates amyloid-beta deposition and improves cognition in the 5XFAD model of amyloid deposition. J Neuroinflammation. 2020;17:238. 32795308 10.1186/s12974-020-01915-0 PMC7427742

[136] Jain N, Lewis CA, Ulrich JD, Holtzman DM. Chronic TREM2 activation exacerbates Abeta-associated tau seeding and spreading. J Exp Med. 2023;220. 10.1084/jem.20220654 PMC9559604 36219197

[137] Pascoal TA, Benedet AL, Ashton NJ, Kang MS, Therriault J, Chamoun M, Savard M, Lussier FZ, Tissot C, Karikari TK, et al. Microglial activation and tau propagate jointly across braak stages. Nat Med. 2021;27:1592–9. 34446931 10.1038/s41591-021-01456-w

[138] Zhao A, Jiao Y, Ye G, Kang W, Tan L, Li Y, Deng Y, Liu J. Alzheimer’s Disease Neuroimaging I: Soluble TREM2 levels associate with conversion from mild cognitive impairment to Alzheimer’s disease. J Clin Invest. 2022;132. 10.1172/JCI158708 PMC9753995 36519540

